# A network analysis of mental, somatic health, and perceived social supports among Chinese pregnant and postpartum women

**DOI:** 10.1080/16549716.2026.2615495

**Published:** 2026-02-02

**Authors:** Rong Li, Li Lu, Zi-Wei Li, Xiao-Dong Qin, Duomei Ren, Huijuan Guang, Baibing Mi, Zhongliang Zhou, Shou Liu, Sha Lai, Qing Shen, Yan Bai

**Affiliations:** a Institute of Health Management and Policy, School of Public Policy and Administration, Xi’an Jiaotong University, Xi’an, China; b System Behavior and Management Laboratory, Philosophy and Social Sciences Laboratory of the Ministry of Education, Xi’an Jiaotong University, Xi’an, China; c Department of Obstetrics, Shaanxi Provincial People’s Hospital, Xi’an, China; d Department of Gynecology, Hanzhong People’s Hospital, Hanzhong, China; e School of Public Health, Xi’an Jiaotong University, Xi’an, China; f Department of Public Health, Medical College, Qinghai University, Xining, China; g Clinical Research Center for Mental Disorders, Shanghai Pudong New Area Mental Health Center, Tongji University School of Medicine, Shanghai, China; h Institute for Advanced Study, Tongji University, Shanghai, China; i Unit of Integrative Epidemiology, Institute of Environmental Medicine, Karolinska Institutet, Stockholm, Sweden; j The Second Affiliated Hospital, Xi’an Medical University, Xi’an, China

**Keywords:** network analysis, mental health, somatic health, support, pregnant and postpartum women

## Abstract

**Background:**

The mental burdens among pregnant and postpartum women were exacerbated by cultural expectations and policy shifts, which can be mitigated by social support.

**Objective:**

To identify the networks of comorbid somatic and mental health, diverse sources of support (interpersonal and policy), and symptom–support interactions in pregnant and postpartum women.

**Methods:**

Participants were recruited from seven Chinese tertiary hospitals. Health conditions, supports, and combined system networks were estimated. Core symptoms and support sources were identified via centrality indices.

**Results:**

Two thousand nine hundred and eighty-nine participants were included, network analysis identified ‘feeling tired/having low energy’ and ‘suicidal thoughts’ in depression symptoms as the most central symptoms. Strongest edges were identified between ‘uncontrollable worry-trouble relaxing’ (anxiety), ‘slowed movement-suicidal thoughts’ (depression), and ‘feeling your heart pound or race-shortness of breath’ (somatic symptoms). Somatic health exhibited strong connections with depression symptoms, especially between ‘trouble sleeping’ and ‘sleep problems’. In the support network, ‘my friends offer practical help’ and ‘fiscal assistance programs’ exhibited highest strength centrality, ‘friends share joys and sorrows with me’ and ‘family willingly helps me make decisions’ were key mediators with high betweenness. All supports were negatively associated with mental/somatic symptoms. Family and friend support had stronger negative connections with hopelessness. ‘Health management’, ‘reliable friends’, and ‘family emotional support’ presented the strongest negative relationships with mental health.

**Conclusion:**

Fatigue and suicidal ideation were identified as interconnected symptoms, informal interpersonal support, and fiscal assistance as key elements. Preventions and interventions should prioritize core symptoms while leveraging the power of support networks, to safeguard maternal health and well-being.

## PAPER CONTENT

- **Main findings:** Fatigue and suicidal ideation were identified as interconnected symptoms in mental health symptoms of pregnant and postpartum women, informal interpersonal support, and fiscal assistance as key elements in the support system.
- **Added knowledge:** This study provided a novel blueprint for the network of mental health among pregnant and postpartum women, pinpointing fatigue and suicidality as core treatment targets and clarifying how distinct support sources function as key mediators.
- **Global health impact for policy and action:** These findings mapped clear imperatives that preventions and interventions should prioritize core symptoms while leverage the power of support networks, to safeguard maternal mental health and well-being.

## Background

While the timeframe of the perinatal period is diversely defined, ranging from the World Health Organization (WHO)’s traditional (28 weeks of pregnancy to 7 days after birth) and updated (from 22 weeks’ gestation) criteria to some researchers’ definition spanning from pregnancy up to 1 year postpartum [1], it is characterized by a process of neural reorganization [2], significant physical and emotional changes, which could entail a far-reaching impact on maternal well-being, infant’s health, and family welfare [3–6]. Within this array of complex challenges, disturbances in somatic and mental health during pregnancy and postpartum emerge as a pivotal concern, which have been globally reported, such as overweight or obesity [7], anxiety [8], depression [8], hopelessness [9], posttraumatic stress disorder [10], sleep disorders [11], and suicide [12]. The prevalence of having at least one type of anxiety disorder of women during the pregnancy and postpartum period was 20.7% [13], and the prevalence of poor sleep quality was 45.7% among pregnant women [14]. A meta-analysis presented a pooled prevalence of any depressive disorder to be 31.4% among pregnant and postpartum women in low- and middle-income countries, with the prevalence of major depressive disorder of 17% [15]. These challenges are particularly pronounced in low-income and middle-income countries, where heightened stress of reproduction, limited access to mental health services and social support to women at childbearing age, as well as socioeconomic disparities may exacerbate the adverse mental health problems [16–18].

In countries like China, traditional culture expectations emphasize idealized motherhood that prioritizes childbearing over career development, imposing substantial somatic and psychological burden on women of reproductive ages [19–21]. Mental and physical health problems were frequently comorbid during pregnancy and postpartum [21], with somatic symptoms serving as essential indicators for assessing depression [22]. For example, women with depression and/or anxiety were more likely to report somatic symptoms compared to those without these conditions [23]. Network analysis further revealed positive connections between mental and somatic symptoms, such as relationship between poor sleep and fatigue in pregnant women, diminished interest in activities and depressed mood, and appetite change and guilt in postpartum women [22]. In addition, among Chinese women at high risk for perinatal depression, perceived stress and hopelessness/suicidality were identified as the most susceptible symptoms within the symptom network [24]. Therefore, exploring the somatic-psychological symptom network in women during pregnancy and postpartum not only advances scientific understanding of comorbid presentations but directly enhances clinical practice, supporting early, integrated, and culturally adapted care for this vulnerable population.

Practical measures targeting to improve somatic and mental health of pregnant and postpartum women have become one of the essential public health priorities. Social support theory suggests that access to supportive relationships or systems, whether through emotional support, tangible assistance, informational guidance, or a sense of belonging, are vital for an individual’s mental, emotional, and physical health, helping people cope with difficulties and acting as a buffer against pressure and stress [25,26]. Support during pregnancy and postpartum comes from a combination of professional medical teams, a personal social network (partners, family, and friends), specialized community and online resources, and human-oriented inclusive policies. Perceived social support played a critically protective role on somatic and mental health during pregnancy and postpartum [27,28], and the diversity of support providers in maternal social network is of great importance when taking family type (e.g. married, single and cohabiting, single) into consideration [27]. For example, the declines in social support and mental health post-delivery were previously demonstrated, and perceived social support during the postpartum period predicted better mental health in women by controlling for social support and mental health prior to the childbirth [29]. Specifically, partner support during childbirth was associated with lower level of postpartum mental health problems [30]. One study among pregnant women revealed that greater social support was associated with better self-rated health, greater sleep quality, fewer health-impairing behaviors [31]. Therefore, optimizing support networks through diversified provision represents one of the most essential strategies for enhancing mental health among pregnant and postpartum women. In addition, the rapid transition from the one-child policy (1979–2016) to the three-child policy (2021) in China [32] has created additional challenges for women, such as delivery at advanced age, repeated cesarean deliveries, and higher risk of neonatal complications [33–35]. Complementing these changes, the government has enacted a series of policies to support healthcare access, parental allowance, financial subsidies, social services, and employment equality to ensure social warfare for these women and their families. The summarized components of support system for pregnant and postpartum women are presented in Supplemental Figure S1. However, how the perceived accessibility of public support was related to somatic and mental health is not fully understood.

Despite extensive research on the relationship between maternal mental health and social support, the networks of symptom–support interactions remain underexplored. And, it should be noted that existing research studying social support in maternal mental health has primarily emphasized family and friend networks, with the mechanisms of policy support receiving less attention. In addition, maternal mental health symptoms, particularly anxiety, depression, hopelessness, and somatic symptoms, etc., were often individually studied, with the networks of substantial comorbidities and overlapped symptoms and their relationships have only gained extensive attention recently and need to be further unravelled.

Network analysis is a data-driven approach that provides a visual depiction of the complex associations among individual symptoms [36]. It allows the identification of highly central symptoms and symptom–symptom interactions, and produces spatially ordered networks [37], which could therefore help us to elucidate complex relationships by revealing relationships, patterns, and structures among mental and somatic health components, and support systems and identify core symptoms within the networks, and to further facilitate the identification of targets for precise prevention and intervention strategies of mental health among pregnant and postpartum women in China. Therefore, this study employs network analyses to (1) identify the networks of comorbid somatic and mental health symptoms in Chinese pregnant and postpartum women, (2) determine diverse sources of support (interpersonal and policy) within social support networks, and (3) examine health symptom–support interactions.

## Methods

### Study design

The data used in the current study were derived from the ‘Study on Maternal Mental Health Management in Western China’, which was conducted annually as a cross-sectional survey every January–February since 2023. Data from the years of 2024 and 2025 were analyzed in this study. The random sampling method was used to recruit pregnant and postpartum women who visited the Department of Obstetrics in seven tertiary hospitals in Qinghai, Shaanxi, and Sichuan provinces. Eligible participants were those who met the following criteria: (1) 18 years old or above and (2) were pregnant during the survey period or had given birth within the past 12 months. We followed the principle of voluntary participation and anonymity in conducting the study, with all participants voluntarily signed the informed consent. The study was approved by the Research Ethics Committee of Xi’an Jiaotong University (No. 2023–1628). Two thousand nine hundred and eighty-nine e-questionnaires (1382 in 2024, and 1607 in 2025) were collected via an online survey platform, which can help to prevent missing data and improve the data quality. Quality control for electronic questionnaires in the current study included screening for duplicate IP addresses and, in the data analysis phase, examining and addressing outliers, logical inconsistencies under standardized criteria, and missing values. Our study was carried out in accordance with the principles of the Declaration of Helsinki.

### Measures

The following sociodemographic characteristics were collected, age at hospital visit (years), ethnicity (Han/others), monthly household income (<5000 CNY/5000–10,000/10,000 and more), educational level of the participant and her partner (primary school or below/middle school/college or above), employment status of the participant and her partner (unemployed/other), antenatal and childbirth status (antenatal/postpartum), number of children (one/two or more; for participants in the prenatal period, including the soon-to-be-born baby).

Anxiety symptoms over the past 2 weeks were evaluated using the validated Chinese version of the 7-item Generalized Anxiety Disorder scale (GAD-7, Cronbach’s alpha: 0.89 [38,39]). Each item is scored from 0 to 3, yielding a total score of 0 to 21, with higher score indicating more severe anxiety symptoms and a score of 10 or above indicating probable anxiety in our study [38].

Depression symptoms experienced over the last 2 weeks were assessed using the validated Chinese version of the 9-item Patient Health Questionnaire (PHQ-9, Cronbach’s alpha: 0.86 [40,41]). Participants indicate how often they had been bothered by each symptom using a 4-point Likert scale ranging from 0 (not at all) to 3 (nearly every day), yielding a total score of 0 to 27. Higher total score indicates greater severity of depression symptoms, participants with a score of 10 or greater were considered as having moderate or severe depression symptoms (probable depression) [41].

Hopelessness level was assessed using the self-reported 20-item Beck Hopelessness Scale (BHS, Cronbach’s alpha: 0.81 [42]), which assesses negative attitudes and expectations about the future [43], each item was scored dichotomously as 0 (indicating the absence of hopelessness) or 1 (presence of hopelessness), with the 9 positively phrased items (e.g. including items 1, 3, 5, 6, 8, 10, 13, 15, and 19) being reverse-scored (R hereafter) and 11 negatively phrased items scored as stated, yielding a total score ranging from 0 to 20. Higher total scores reflect greater levels of hopelessness.

Somatic symptoms experienced by the participants over the last 4 weeks were assessed using the 15-item Patient Health Questionnaire (PHQ-15, Cronbach’s alpha: 0.81 [44,45]). To be noted, given that menstrual-related issues are not applicable for pregnant and postpartum women (depending on the individual situation for the latter), the item indicating ‘menstrual cramps or other problems with your periods’ was excluded from the questionnaire. The score of each item ranged from 0 (not bothered at all) to 2 (bothered a lot), yielding a total score of 0 to 28. Higher total score indicates greater severity of somatic symptom burden.

Participants’ perceived social support (Table 1: Social support 1–12, SU1-SU12) was assessed using the 12-item Multidimensional Scale of Perceived Social Support [46] (MPSSS, Cronbach’s alpha: 0.93 [40]), which helps to measure the perceived social support from three sources: significant other (SU1-SU4), family (SU5-SU8), and friend (SU9-SU12). Each item on the MSPSS was rated on a 7-point response from 1 (very strongly disagree) to 7 (very strongly agree), with total score ranging from 12 to 84, and total score of each subscale ranges from 4 to 28. Higher score represents higher levels of perceived social support.

**Table 1.** Basic information of anxiety, depression, somatic, hopelessness symptoms, and social support.

Label	Item	Mean (SD)	Label	Item	Mean (SD)
PHQ15-1	Stomach pain	0.33 (0.51)	BHS5	Enough time to achieve goals (R)	0.46 (0.50)
PHQ15-2	Back pain	0.46 (0.60)	BHS6	Expect future success (R)	0.24 (0.43)
PHQ15-3	Pain in your arms, legs, or joints	0.43 (0.60)	BHS7	Future seems dark	0.12 (0.32)
PHQ15-5	Headaches	0.31 (0.51)	BHS8	Expect more good things than others (R)	0.25 (0.43)
PHQ15-6	Chest pain	0.22 (0.44)	BHS9	Bad luck, no hope for change	0.16 (0.37)
PHQ15-7	Dizziness	0.29 (0.48)	BHS10	Past prepared me for the future (R)	0.52 (0.50)
PHQ15-8	Fainting spells	0.09 (0.32)	BHS11	Only unpleasantness ahead	0.13 (0.34)
PHQ15-9	Feeling your heart pound or race	0.35 (0.53)	BHS12	Won’t get what I truly want	0.17 (0.37)
PHQ15-10	Shortness of breath	0.39 (0.54)	BHS13	No use trying (likely to fail)	0.28 (0.45)
PHQ15-11	Pain or problems during sexual intercourse	0.17 (0.41)	BHS14	Future feels vague/uncertain	0.14 (0.35)
PHQ15-12	Constipation, loose bowels, or diarrhea	0.49 (0.61)	BHS15	Future will be better than now (R)	0.24 (0.43)
PHQ15-13	Nausea, gas, or indigestion	0.44 (0.57)	BHS16	Strong faith in the future (R)	0.14 (0.35)
PHQ15-14	Feeling tired or having low energy	0.55 (0.62)	BHS17	Never get desires, so wanting is foolish	0.16 (0.36)
PHQ15-15	Trouble sleeping	0.54 (0.65)	BHS18	Unlikely to find satisfaction	0.19 (0.39)
GAD1	Nervousness or anxiety	0.48 (0.68)	BHS19	More good times than bad ahead (R)	0.35 (0.48)
GAD2	Uncontrollable worry	0.34 (0.61)	BHS20	No sense trying (failure inevitable)	0.14 (0.34)
GAD3	Generalized worry	0.43 (0.66)	SU1	There are people I can rely on when I have problems.	4.70 (2.59)
GAD4	Trouble relaxing	0.39 (0.63)	SU2	Share joys and sorrows with certain people.	4.74 (2.64)
GAD5	Restlessness	0.25 (0.55)	SU3	People truly comfort me during difficulties.	4.78 (2.63)
GAD6	Irritability	0.44 (0.67)	SU4	There are people who care about my feelings.	4.79 (2.49)
GAD7	Fear of horrible events	0.30 (0.60)	SU5	My family provides concrete assistance when needed.	5.10 (2.60)
PHQ9-1	Little interest in activities	0.36 (0.64)	SU6	I receive emotional help and support from my family.	5.19 (2.58)
PHQ9-2	Feeling depressed/hopeless	0.34 (0.60)	SU7	I can discuss my problems with family.	5.02 (2.59)
PHQ9-3	Sleep problems	0.51 (0.72)	SU8	My family willingly helps me make decisions.	5.02 (2.57)
PHQ9-4	Low energy	0.52 (0.70)	SU9	My friends offer practical help.	4.94 (2.33)
PHQ9-5	Poor appetite or overeating	0.44 (0.68)	SU10	I can rely on friends in tough situations.	5.01 (2.37)
PHQ9-6	Self-blame/guilt	0.27 (0.58)	SU11	I can talk to friends about my troubles.	4.86 (2.45)
PHQ9-7	Poor concentration	0.29 (0.59)	SU12	Friends share joys and sorrows with me.	4.78 (2.46)
PHQ9-8	Slowed/restless movement	0.22 (0.53)	SU13	Health Management Policy	4.47 (4.28)
PHQ9-9	Suicidal thoughts	0.18 (0.49)	SU14	Time Support Policy	4.60 (3.91)
BHS1	Feel hopeful about the future (R)	0.20 (0.40)	SU15	Fiscal Assistance Program	4.07 (4.56)
BHS2	Want to give up (nothing can improve)	0.25 (0.43)	SU16	Public Service Delivery Framework	4.63 (4.43)
BHS3	Believe bad situations will get better (R)	0.58 (0.49)	SU17	Employment Security Policy	3.43 (3.92)
BHS4	Cannot imagine life in 10 years	0.44 (0.50)	–	–	–

Note: SD, Standard deviation. (R) = Reverse-scored (score 1 point for ‘false’).

The accessibility of the Government’s fertility support policies (SU13–SU17) was collected using the self-designed 22-item questionnaire across five dimensions (Supplemental Table S1): health management (with six items), time support (four items), financial assistance (six items), services provision (three items), and employment protection (three items) [47]. Cronbach’s alpha was 0.94 in the current study. Participants were asked to indicate which fertility support policies they know were available to access by selecting the option of ‘yes’ or ‘no’. Each ‘yes’ response was counted as 1 point, allowing the self-assessment questionnaire to quantify participants’ potential benefits that they can get from the government-supported fertility policies on a numerical scale. The accessibility of the Government’s fertility support policies was quantified as the sum of ‘yes’ responses (range: 0–22) and rescaled to 1–7 for consistency with the MSPSS metric. Higher scores indicate more extensive accessibility of available fertility support policies.

The original items and their briefly described version for social support and other health-related items are presented in Table 1.

### Statistical analysis

The mean and standard deviation (SD) were calculated to describe anxiety symptoms, depression symptoms, somatic symptoms, hopelessness, and support from family, friends, and significant others. We employed a Gaussian Graphical Model (GGM) to construct and analyze three distinct networks to explore the relationships between mental health problems and somatic health, perceived supports, and their interactions among pregnant and postpartum women. Guided by social support theory, we initiated to incorporate the dimension of health and social support, and their interactions. This sequential strategy provided a more nuanced interpretation than a single or complex model, as it separated connections within communities from those between them. The networks were constructed as follows:

- **Network 1:** The network of mental (depression, anxiety symptoms, and hopelessness) and somatic health among pregnant and postpartum women.
- **Network 2:** The network of perceived supports (support from the significant others, families, friends, and government fertility support).
- **Network 3:** Comprehensive network of mental, somatic health, and perceived supports.

The network structures based on Spearman correlation coefficients were estimated applying the LASSO regularization and visualized using Fruchterman–Reingold algorithm [48], where nodes represent mental and physical health symptoms/syndromes/domains or support sources in the current study, the thickness and color of edges represent the strength and positive/negative association of these relationships between symptoms. To eliminate spurious correlations, we selected the optimal regularization parameter using the Extended Bayesian Information Criterion (EBIC), the tuning parameter *γ* (gamma) for the EBIC was set to 0.5 [49].

The centrality indices include strength centrality, closeness centrality, betweenness centrality, expected influence (EI) for network 1 and 2 and bridge EI for network 3, all of which serve to assess the significance of nodes within the network, with higher centrality scores indicating a more pivotal role of the symptoms. Specifically, strength centrality indicates the degree to which each node is connected to other nodes in the network, closeness centrality quantifies how close a node is to all other nodes, and betweenness centrality measures a node’s influence by assessing how often a node lies on the shortest paths between other nodes, a higher betweenness centrality indicates that the node is serving as a key mediator in the connections of symptoms.

The stability of centrality indices of the generated network structure was evaluated using the correlation stability coefficient (CS-coefficient) [50]. Consistently high centrality indices across bootstrap samples, with a CS-coefficient above 0.5 [50], illustrate the network structure is stable and reliable for accurate interpretation. Network models were fitted using the R package ‘bootnet’. To assess the stability of network edges, 95% confidence intervals (CIs) for each edge weight were calculated by employing a nonparametric bootstrap method with 1000 iterations, with narrow CIs indicating good reliability of the estimated edge weights.

Sensitivity analysis excluding the community of hopelessness to construct the network (network 1 and 3), and subgroup group analysis by household income (network 1, 2 and 3) were further conducted. All data were analyzed in R version 4.5.0 via RStudio, with a significant *α* threshold of 0.05 (two-tailed).

## Results

### Descriptive statistics

A total of 2989 participants were included, with an average age of 30.6 (SD: 5.33) years, and 69.9% were pregnant women (*n* = 2090). The basic characteristics of the included participants are shown in Supplemental Table S2. The prevalence of probable depression and probable anxiety were 7.39% (95% CI: 6.46–8.33) and 5.52% (95% CI: 4.70–6.34), respectively (Supplemental Table S1). The mean and SD of all items in each mental or somatic health symptoms, and support scales are presented in Table 1. The total score for depression symptoms, anxiety symptoms, hopelessness, and somatic symptoms was 3.14 (SD: 4.40), 2.60 (SD: 3.70), 5.16 (SD: 3.50), and 5.04 (SD: 4.90), respectively. Regarding perceived social support, participants reported a mean score of 4.75 (SD: 5.95), 5.08 (SD: 6.11) and 4.90 (SD: 5.93) for support from significant others, family, and friends, respectively, the corresponding level of government support was 4.24 (SD: 8.29).

### Network analyses

**Figure 1.**
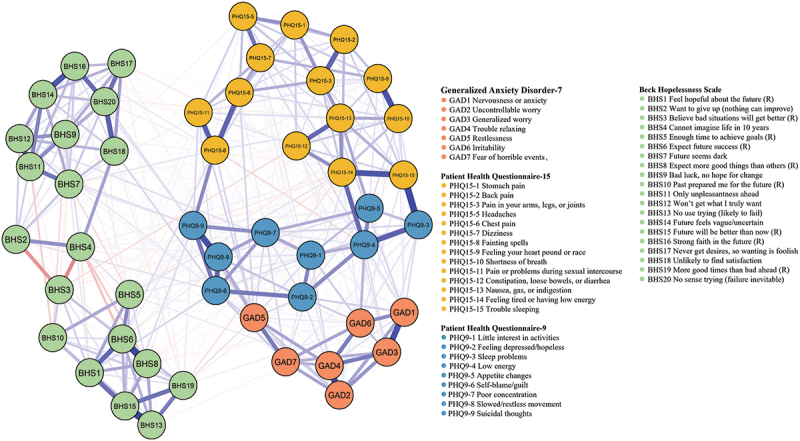
Symptom network of somatic, depression, anxiety symptoms and hopelessness among Chinese pregnant and postpartum women. Note: Red lines represent negative correlations, blue lines represent positive correlations.

**Figure 2.**
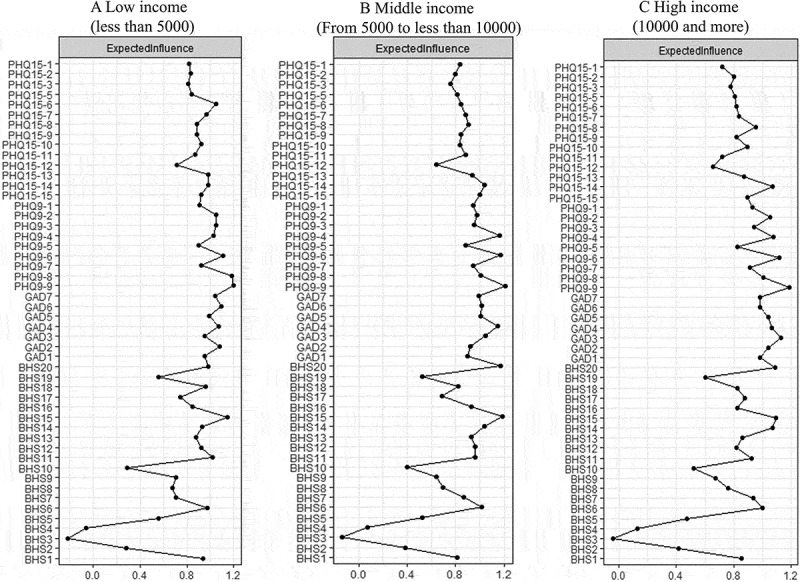
Centrality measures of three networks.

Network 1: The network structure of mental and somatic health symptoms is illustrated in Figure 1. The strongest edge in the anxiety symptom subnetwork was between ‘uncontrollable worry’ (GAD2) and ‘trouble relaxing’ (GAD4), followed by the connections between ‘nervousness or anxiety’ (GAD1) and ‘generalized worry’ (GAD3). The strongest edges in the depression symptoms and hopelessness subnetwork were observed between ‘slowed movement’ (PHQ9-8) and ‘suicidal thoughts’ (PHQ9-9), and between ‘expect future success (R)’ (BHS6) and ‘expect more good things than others (R)’ (BHS8), respectively. In the somatic symptom network, the strongest edges were found between ‘feeling your heart pound or race’ (PHQ15-9) and ‘shortness of breath’ (PHQ15-10), and between ‘feeling tired or having low energy’ (PHQ15-14) and ‘trouble sleeping’ (PHQ15-15). Somatic health symptoms exhibited strong connections with depression symptoms, especially between ‘trouble sleeping’ (PHQ15-15) and ‘sleep problems’ (PHQ9-3). The centrality indices of Network 1 are shown in Figure 2 and Supplemental Figure S2. The highest EI was identified in the node of ‘suicidal thoughts’ (PHQ9-9), ‘self-blame’ (PHQ9-6), and ‘future will be better than now’ (BHS-15). The node of ‘feeling tired or having low energy’ (PHQ15-14) presented the highest strength value of 1.39, followed by ‘expect future success (R)’ (BHS6) and ‘suicidal thoughts’ (PHQ9-9) with the values of 1.35 and 1.33, respectively. ‘Self-blame/guilt’ (PHQ9-6), ‘feeling tired or having low energy’ (PHQ15-14), and ‘fainting spells’ (PHQ15-8) were identified as key mediators.

**Figure 3.**
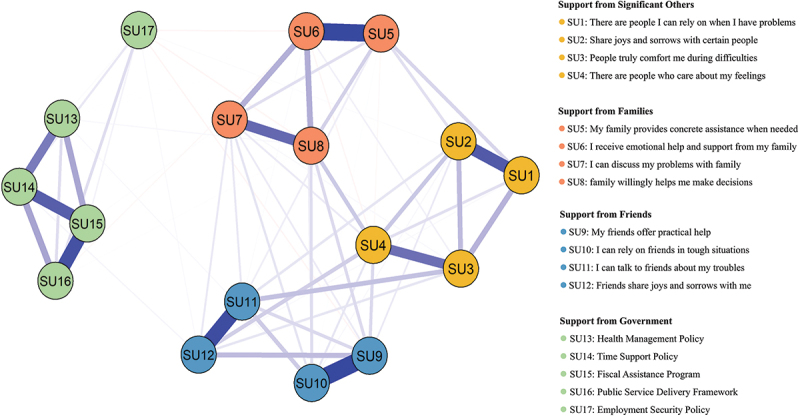
Symptom network of perceived supports. Note: Red lines represent negative correlations, while blue lines represent positive correlations.

Network 2: The network structure of perceived supports is depicted in Figure 3. The node with highest strength (Supplemental Figure S3) and EI value (Figure 2) were identified in ‘my friends offer practical help’ (SU9; strength value: 1.16), followed by ‘fiscal assistance program’ (SU15; strength value: 1.15) and ‘I receive emotional help and support from my family’ (SU6; strength value: 1.13). Betweenness centrality indicated that ‘friends share joys and sorrows with me’ (SU12; betweenness value: 52) and ‘family willingly helps me make decisions’ (SU8; betweenness value: 48) served as key mediators in the network (Supplemental Figure S3).

**Figure 4.**
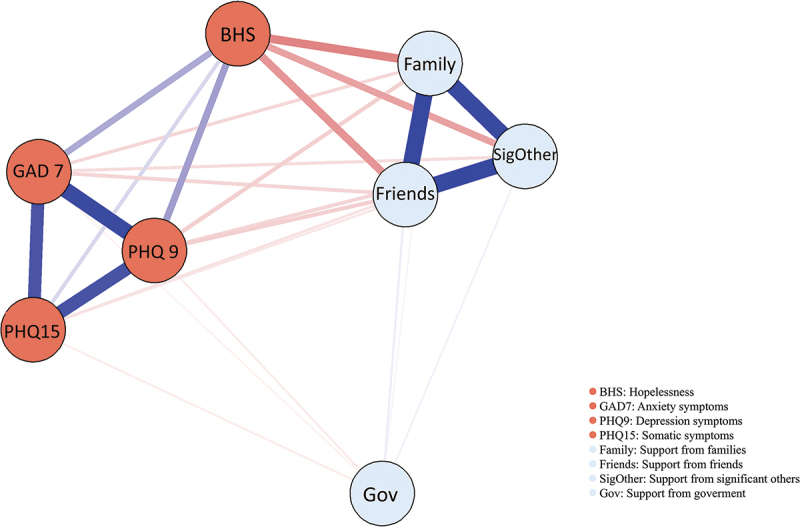
Network of mental health problems and perceived supports.

Network 3: In the comprehensive network structure (Figure 4), all support-related indicators (support from families, friends, significant others, and government) presented negative connections with mental and somatic health indicators (anxiety and depression symptoms, hopelessness and somatization). Specifically, anxiety, depressive, and somatic symptoms were most closely interconnected, while hopelessness presented weaker connections than other indicators. Support from families, friends, and significant others were tightly connected, while support from the government showed weaker connections with these three support indicators. The analysis revealed that friend support and depression were the nodes with the strongest bridge expected influence in the network (Bridge Expected Influence: 1.05; 1.11) (Figure 2).

Regarding the connections between support and health indicators, the node of ‘support from friends’ and ‘support from family’ exhibited stronger negative connections with ‘hopelessness’ than ‘support from significant others’, while ‘support from government’ exhibited the weakest connections. The centrality indices are shown in Supplemental Figure S4. The comprehensive network structure between all support items and health indicators is illustrated in Supplemental Figure S5. ‘Health management’ (SU13), ‘I can rely on friends in tough situations’ (SU10) and ‘I receive emotional help and support from my family’ (SU6) exhibited strong negative connections with hopelessness. The centrality indices are shown in Supplemental Figure S6.

The edge weights of all three networks are presented in Supplemental Tables S4–S6. The results of the stability of edge weights are presented in Supplemental Figures S7A, S8A, and S9A. The 95% CIs for edge weights were narrow, indicating accurate assessment of edge weights. The stability of centrality indices across all networks was evaluated through stability analysis and accuracy assessment (Supplemental Figures S7B, S8b, and S9B). For Network 1 and Network 3, the CS coefficients were higher than 0.5 across all centrality indices indicating good stability of centrality indices. While Network 2 demonstrated a CS coefficient of lower than 0.5 especially for betweenness centrality, indicating a poor stability of betweenness centrality.

### Sensitivity analysis

The sensitivity analysis of Network 1 constructed by excluding the ‘hopelessness’ community, presented the weakest overall connectivity in the estimated network (Supplemental Figures S10 and S11). While the accuracy and stability indices of network 1 and network 3 were not substantially changed, confirming the robustness of our primary network model (Supplemental Figures S11, S12, S13, and S14).

### Subgroup analysis

Subgroup analyses of Networks 1 and 2 by household income showed that PHQ9-9 and SU15 presented the highest EI in all three income groups, respectively (Supplemental Figure S15). Compared to middle- and low-income groups (both 0.95), SU12 had a greater impact in the high-income group (1.03). Network 3 revealed that friend support and depression consistently exhibited the highest BEI across all three income groups (Supplemental Figures S15–S21).

## Discussion

This study employed network analysis to investigate the complex interconnections between mental and somatic health and social supports in Chinese pregnant and postpartum women. Our findings revealed that within the network of mental and somatic health, ‘feeling tired or having low energy’ (PHQ15-14), ‘expect future success (R)’ (BHS6) and ‘suicidal thoughts’ (PHQ9-9) occupied central network positions; and in the network of social support, the ‘my friends offer practical help’ (SU9) and ‘fiscal assistance program’ (SU15) exhibited the highest centrality. Importantly, all sources of support showed negative associations with mental health problems, specifically ‘I receive emotional help and support from family’ (SU6) and ‘I can rely on friends in tough situations’ (SU10) demonstrated a significantly stronger negative association with hopelessness than support from significant other or government, which underscored the importance of support from friends and family in promoting the mental health of this vulnerable group.

Our study found that ‘feeling tired or having low energy’ (PHQ15-14), ‘expect future success (R)’ (BHS6) and ‘suicidal thoughts’ (PHQ9-9) collectively constituted the central structure of the somatic-mental health network, which aligned partially with the previous study indicating perceived stress and hopelessness/suicidality as the most susceptible symptoms using cross-lagged panel network analysis in Chinese high-risk women during the perinatal period [24]. Although we were unable to explore the longitudinal relationships, we revealed the critical triad of a ‘Fatigue-Hopelessness-Suicidality’ pathway presenting the significant vulnerability of women during this special phase. First, pregnant and postpartum women experienced substantial challenges in navigating identity transition and maternal role adaptation [51], with exhaustion stemming from physiological changes, hormonal fluctuations and sleep deprivation [11,52,53], which directly contribute to postpartum depression [53]. Second, the hub position of ‘expectation of future success’ highlighted that the loss of hope was a pivotal psychological mechanism. The challenges of increased responsibilities, loss of freedom, and changed body image could lead to maladaptation and intense feelings of loss and helplessness [51,54]. Crucially, the resource drain caused by fatigue may undermine women’s capacity to envision a positive future. As previously identified, when hope diminished, suicidal ideation became more prominent, reflecting the well-documented role of hopelessness as a strong predictor of suicide [55]. Third, the centrality of suicidal ideation was of particular concern among pregnant women [56], which can lead to maternal mortality [57]. The significant physiological and hormonal changes among pregnant and postpartum women [52] increased the risk of emotional instability and depression, which were identified as strong predictors of suicide [51,58]. Furthermore, the impairment of emotional regulation and stress-coping abilities resulting from newborn care-related sleep deprivation [59,60], consequently lowered the threshold for suicidal ideation among postpartum women.

Our findings align with the main effect model of social support theory, which posits that support networks foster a general sense of belonging, stability, and self-worth, thereby enhancing resilience and buffering against distress across diverse situations [25]. Another key finding also indicates all types of social supports demonstrating negative associations with mental health problems, reinforcing the well-established perspective that social support serves as a protective factor for psychological well-being [25,61].

We further revealed a distinct hierarchical efficacy gradient within the support ecosystem. Informal supports were actively and directly engaged in buffering mental health symptoms, whereas formal governmental supports remained relatively siloed despite its structural centrality. This was consistent with the universal phenomenon in collectivist-oriented societies, individuals are more likely to seek help from family and social networks rather than professional institutions [62]. Informal interpersonal supports were with the merits of accessibility, cost-effectiveness, and free from waiting times, while eligibility restrictions or geographic constraints characterized formal support services [63]. Previous study has demonstrated that informal social support, especially partner support, served as the most critical protective factor against prenatal depression [64]. Supports from other family members, such as parents and in-laws, also played a significant role in alleviating maternal mental health risks [65]. However, our study identified ‘fiscal assistance program’ (SU15) as the second strongest node in the support network, underscoring the critical role of financial support for participants who were under economic instability. It should be noted that Network 2, especially for betweenness centrality, demonstrated a CS coefficient below 0.5; therefore, the findings should be interpreted with caution. This finding highlights the complementary nature of formal and informal support systems, as formal support provided professional expertise and systematic safeguards that informal networks struggled to deliver consistently [63].

The network structure provided clear guidance for prevention intervention strategies, given the centrality of suicidal ideation and fatigue, it suggested that interventions targeting these core symptoms could have cascading benefits across the entire mental health network. Early identification and intervention on core symptoms should be prioritized in mental health screening programs on pregnant and postpartum women. In addition, previous studies have indicated that exercise [66], specific information–motivation–behavioral skills interventions [67], and digital health interventions (DHI), especially when it delivered psychotherapy, such as cognitive-behavioral therapy, interpersonal therapy, or mindfulness [68] significantly reduced diverse mental health conditions on pregnancy and postpartum women, which could be considered. The tiered effectiveness observed across support types suggested that interventions should leverage informal support networks while strengthening formal support systems, progressing toward inclusive goals on dual fronts. Family and friend support networks, especially support from husband [69] and peer-support [70], should be actively incorporated into mental health promotion practices. To be noted, policy makers should consider to integrate psychosocial elements and improve accessibility of formal support services to establish comprehensive support ecosystems.

This study possesses several notable strengths. First, the multicenter design across seven tertiary hospitals in three western Chinese provinces ensured reliable representativeness and generalizability. Second, the Gaussian Graphical Models (GGM) within a network theory framework were innovatively applied, providing novel structural insights into the complex interplay between mental health and social support that traditional methods might be unreachable. This approach successfully identified core symptoms such as suicidal ideation and fatigue within the mental health network and pivotal nodes like fiscal assistance within the support network, along with their critical bridging functions. And, the reliability was ensured by the employment of validated and standardized instruments such as LASSO regularization with EBIC helping to control spurious associations, bootstrap methods to assess edge weight accuracy, and CS coefficients to evaluate centrality stability. Third, this study simultaneously incorporated multidimensional health symptoms and diverse support sources, including the innovative quantification and integration of government policy into the network analysis, offering a holistic perspective.

Some limitations should be acknowledged. First, the cross-sectional nature of the data prevented establishing causal relationships between mental health issues and various sources of support, and the potential meditation role of social support in the relationship between somatic and psychological symptoms should be further studied with available longitudinal data. Second, the measurements for symptoms and perceived supports were self-reported, which could introduce recall biases, especially the application of self-developed ‘Government’s fertility support’ scale could partially pose challenges to the measurement’s scientific rigor and comparability across studies. Third, although this study was multicenter based, the sample was recruited solely from tertiary hospitals, limiting its representativeness for community-based, rural, remote, or nonhelp-seeking populations across China. In addition, we failed to capture the perceived adequacy, quality, and satisfaction levels in terms of perceived government supports, which should be considered in future studies.

## Conclusions

In conclusion, taking advantage of network analyses, this study provided crucial insights in the landscape of mental health and perceived supports for pregnant and postpartum women in China. The identification of fatigue and suicidal ideation as interconnected symptoms within the mental health network, along with informal interpersonal support and fiscal assistance as key elements in the support network, underscored the importance of interpersonal supports, and financial aids for this population. These findings mapped clear imperatives that preventions and interventions should prioritize core symptoms like suicidality and fatigue, while actively strengthen and leverage the power of informal support networks, especially their concrete emotional and instrumental functions. Governmental and institutional support policies should also consider to integrate psychosocial elements, enhance psychological effectiveness and improve accessibility, to safeguard maternal mental health and well-being in China.

## Supplementary Material

Supplementary_clean.docx

STROBE.doc

## Data Availability

The data that support the findings of this study are available from the corresponding author, L. Lu, upon reasonable request.
