# Supplementary material for: A network analysis of mental, somatic health, and perceived social supports among Chinese pregnant and postpartum women
Source: Glob Health Action. 2026 Feb 2;19(1):2615495. doi: 10.1080/16549716.2026.2615495 (PMC12865834; doi:10.1080/16549716.2026.2615495)
Supplement: Supplementary_clean.docx [file ZGHA_A_2615495_SM8557.docx]

**Supplemental Information**

| **Supplemental Figure 1** | Components of the support system among pregnant and postpartum women |
| --- | --- |
| **Supplemental Figure 2** | Centrality measures of network 1 |
| **Supplemental Figure 3** | Centrality measures of network 2 |
| **Supplemental Figure 4** | Centrality measures of network 3 |
| **Supplemental Figure 5** | The network of all items from support, mental and somatic health problems |
| **Supplemental Figure 6** | Centrality measures of network of all items from support, mental and somatic health problems |
| **Supplemental Figure 7** | The accuracy (A) and stability indices (B) of network 1 |
| **Supplemental Figure 8** | The accuracy (A) and stability indices (B) of network 2 |
| **Supplemental Figure 9** | The accuracy (A) and stability indices (B) of network 3 |
| **Supplemental Figure 10** | The network of mental health problems (Network 1) excluding the community of hopelessness |
| **Supplemental Figure 11** | The network of mental health problems and perceived supports (Network 3) excluding the community of hopelessness |
| **Supplemental Figure 12** | Centrality measures of network 1 excluding the community of hopelessness |
| **Supplemental Figure 13** | The accuracy (A) and stability indices (B) of Network 1 excluding the community of hopelessnes |
| **Supplemental Figure 14** | The accuracy (A) and stability indices (B) of Network 3 excluding the community of hopelessness |
| **Supplemental Figure 15** | Networks of mental health and social support by income level |
| **Supplemental Figure 16** | Centrality measures of networks of mental health by income level |
| **Supplemental Figure 17** | The accuracy and stability indices of networks of mental health by income level |
| **Supplemental Figure 18** | Centrality measures of networks of social support by income level |
| **Supplemental Figure 19** | The accuracyand stability indices of networks of social support by income level |
| **Supplemental Figure 20** | Centrality measures of network of mental health problems and perceived supports by income level |
| **Supplemental Figure 21** | The accuracy and stability indices of networks of mental health problems and perceived supports by income level |
| **Supplemental Table 1** | Fertility Support Policies in detail |
| **Supplemental Table 2** | Basic characteristic of the included participants |
| **Supplemental Table 3** | The prevalence of depressive symptoms and anxiety symptoms |
| **Supplemental Table 4** | Edge weight of Network 1 |
| **Supplemental Table 5** | Edge weight of Network 2 |
| **Supplemental Table 6** | Edge weight of Network 3 |

**Supplemental Figure 1** Components of the support system among pregnant and postpartum women


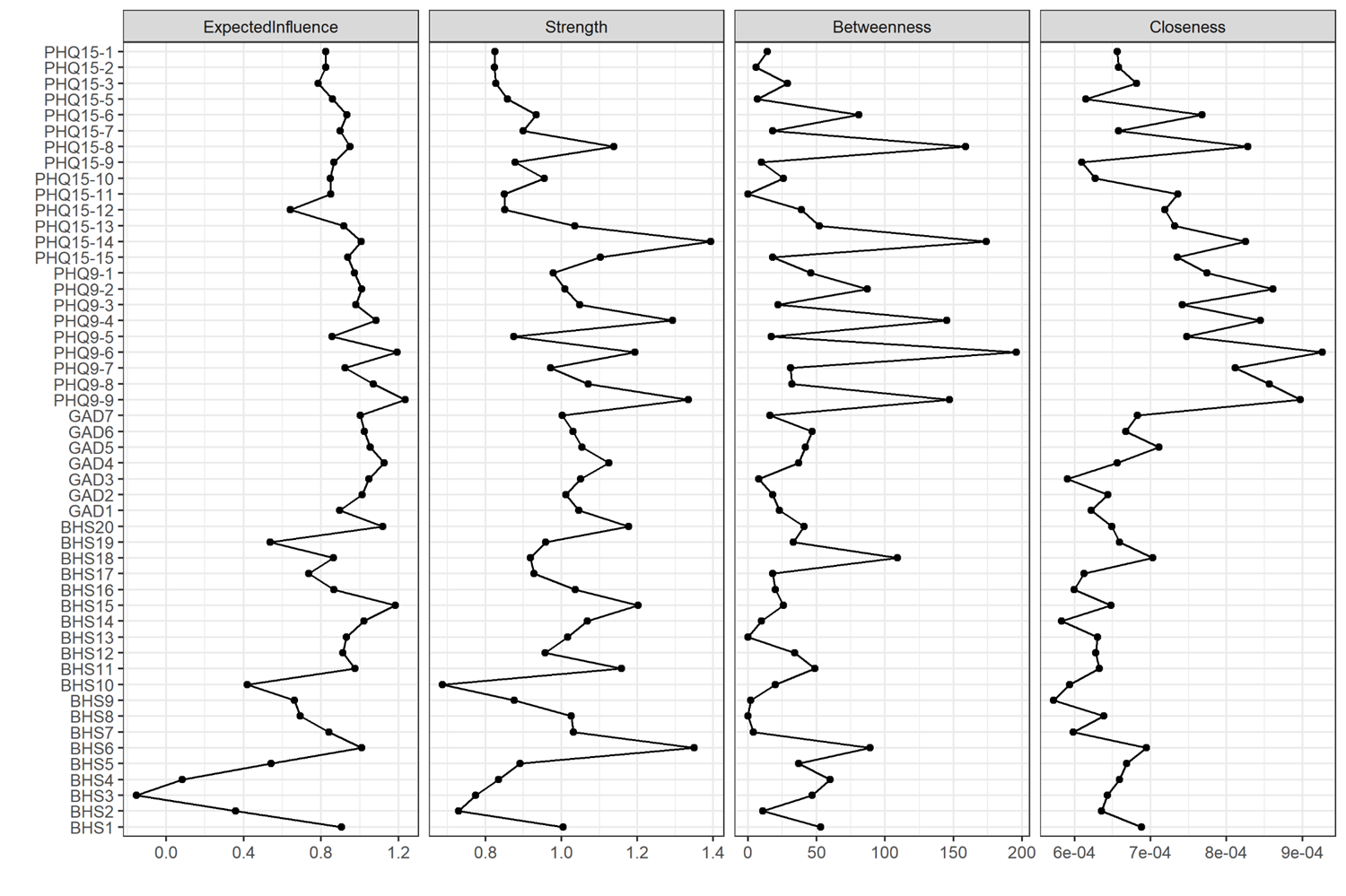


**Supplemental Figure 2** Centrality measures of network 1


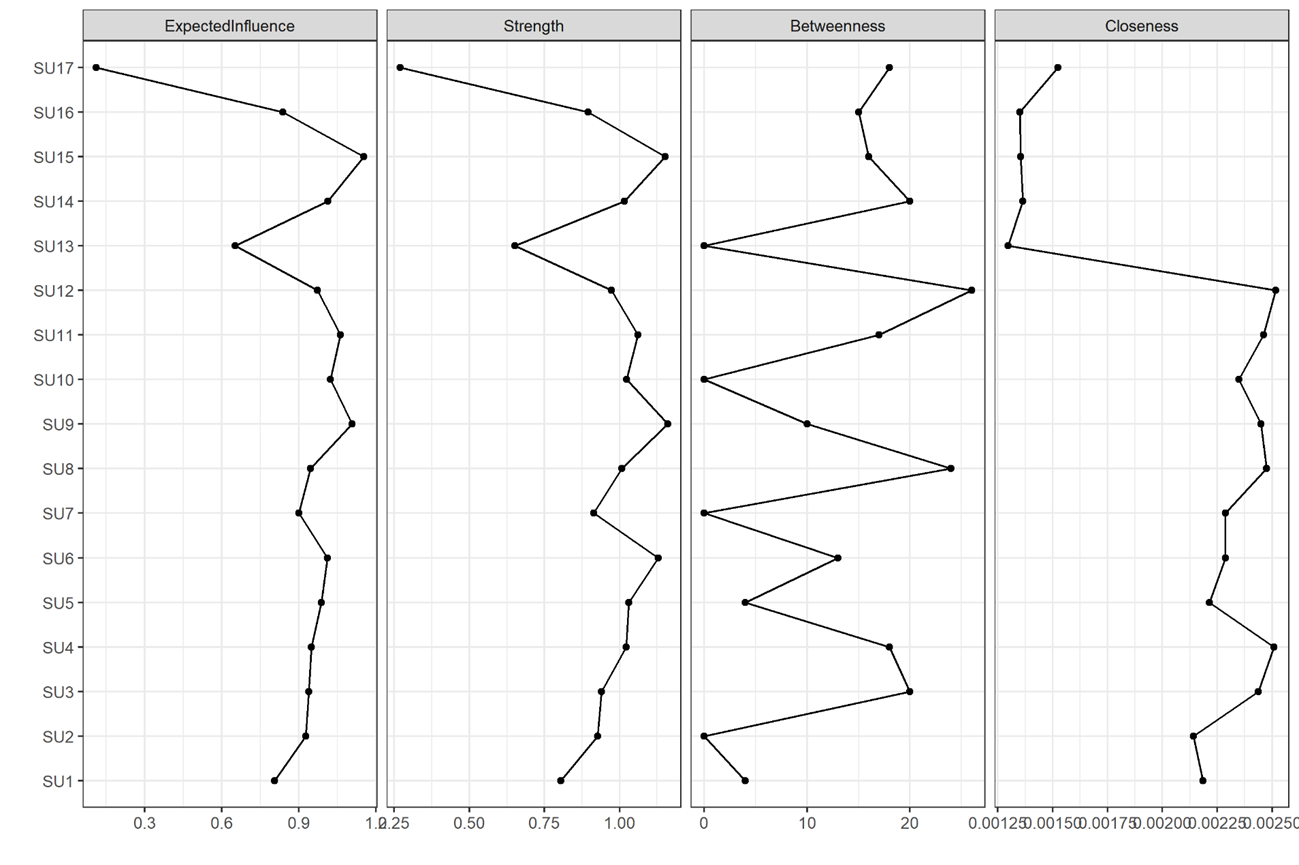


**Supplemental Figure 3** Centrality measures of network 2


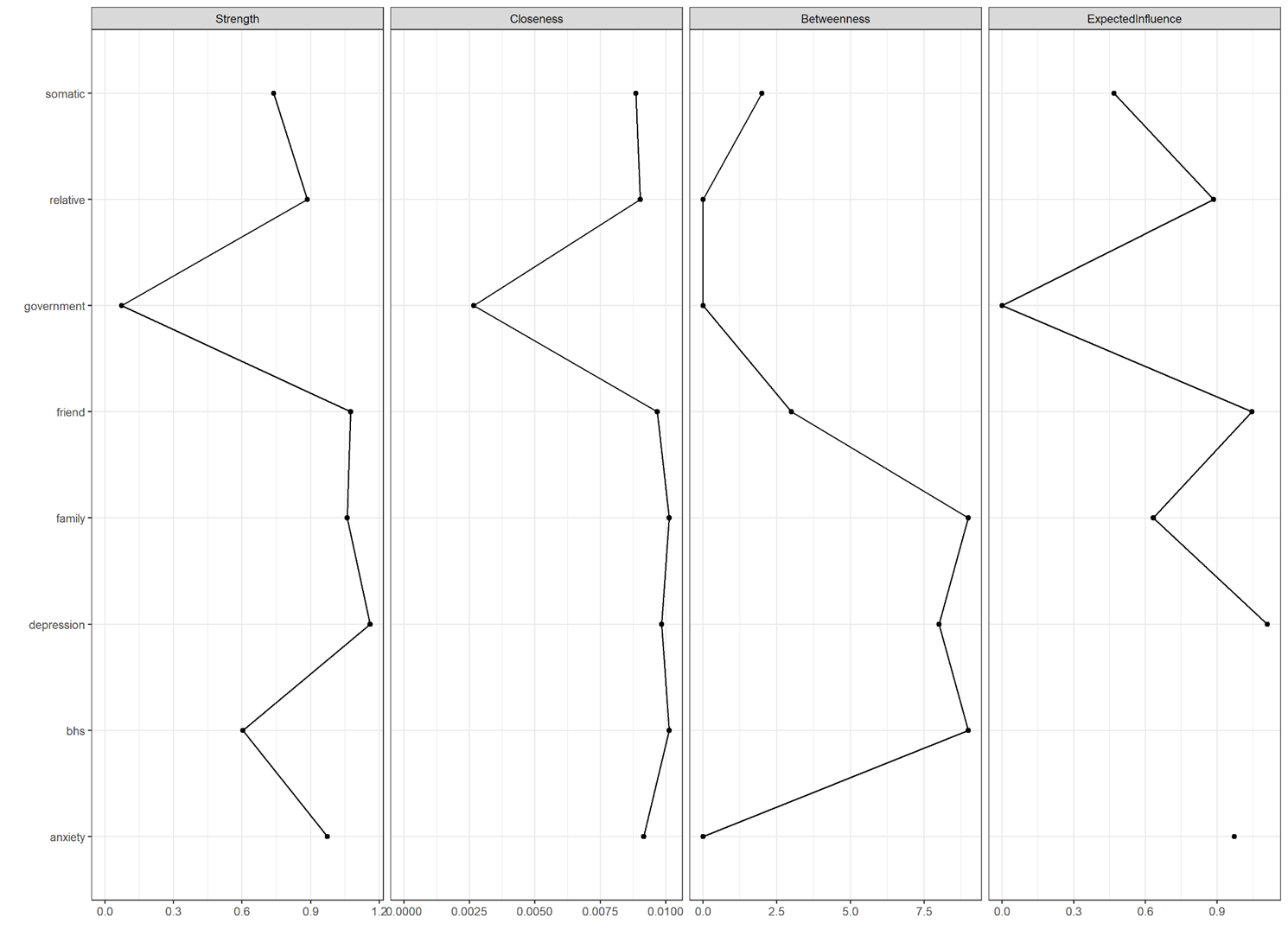


**Supplemental Figure 4** Centrality measures of network 3


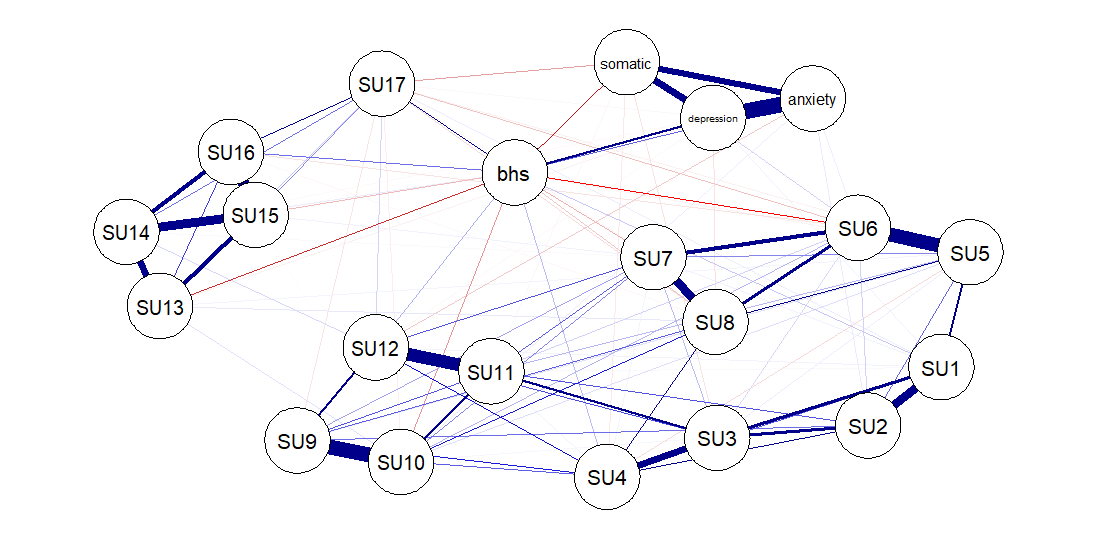


**Supplemental Figure 5** The network of all items from support, mental and somatic health problems


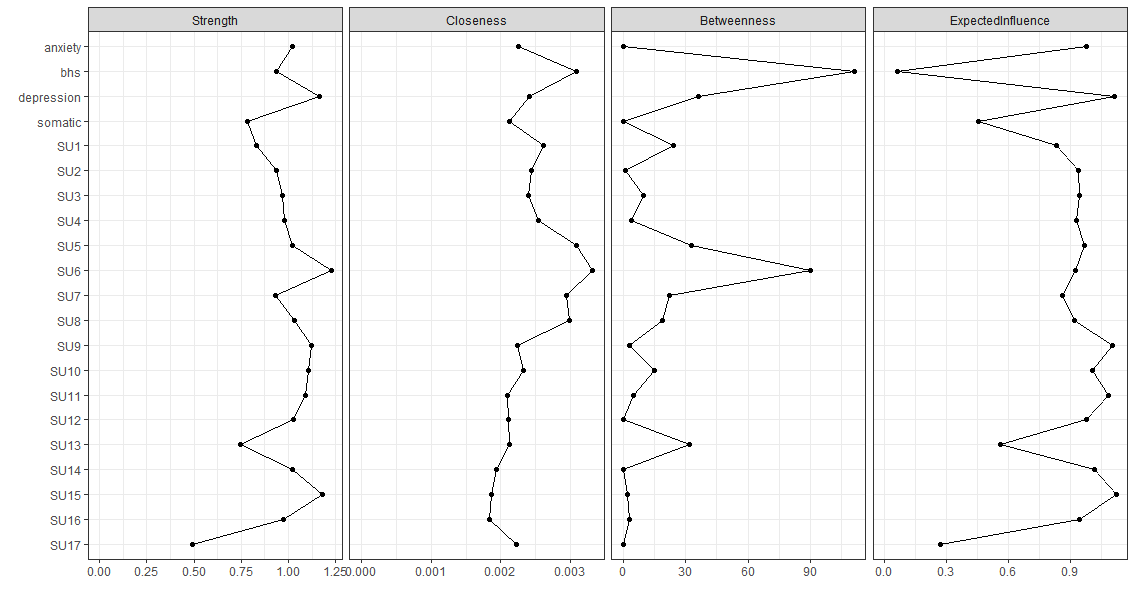


**Supplemental Figure 6** Centrality measures of network of all items from support, mental and somatic health problems


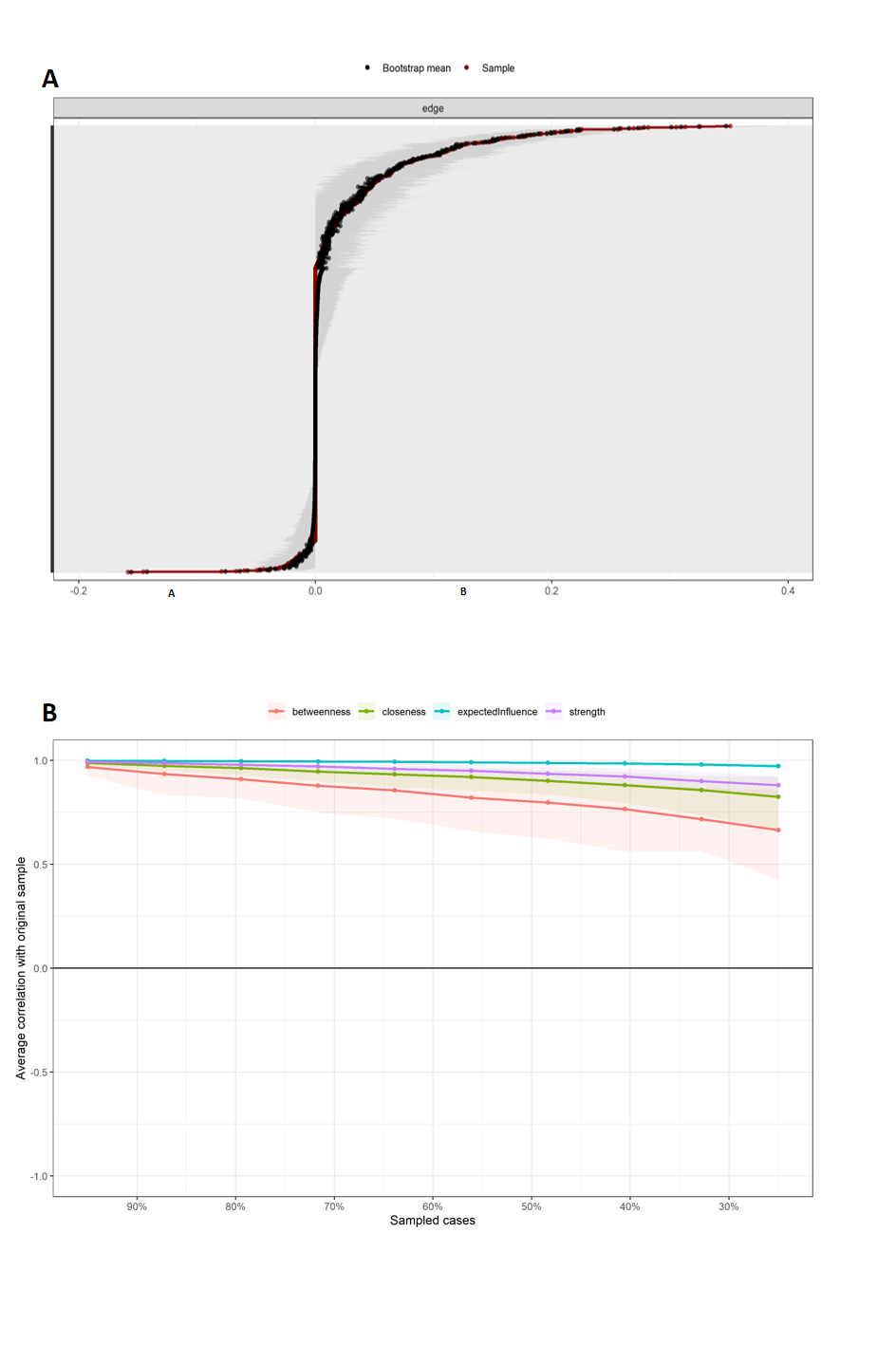


Note: Black lines represent the bootstrapped mean edge weights, red lines represent the edge weights in the study sample. The gray area represents the bootstrap 95% confidence interval.

**Supplemental Figure 7** The accuracy (A) and stability indices (B) of network 1


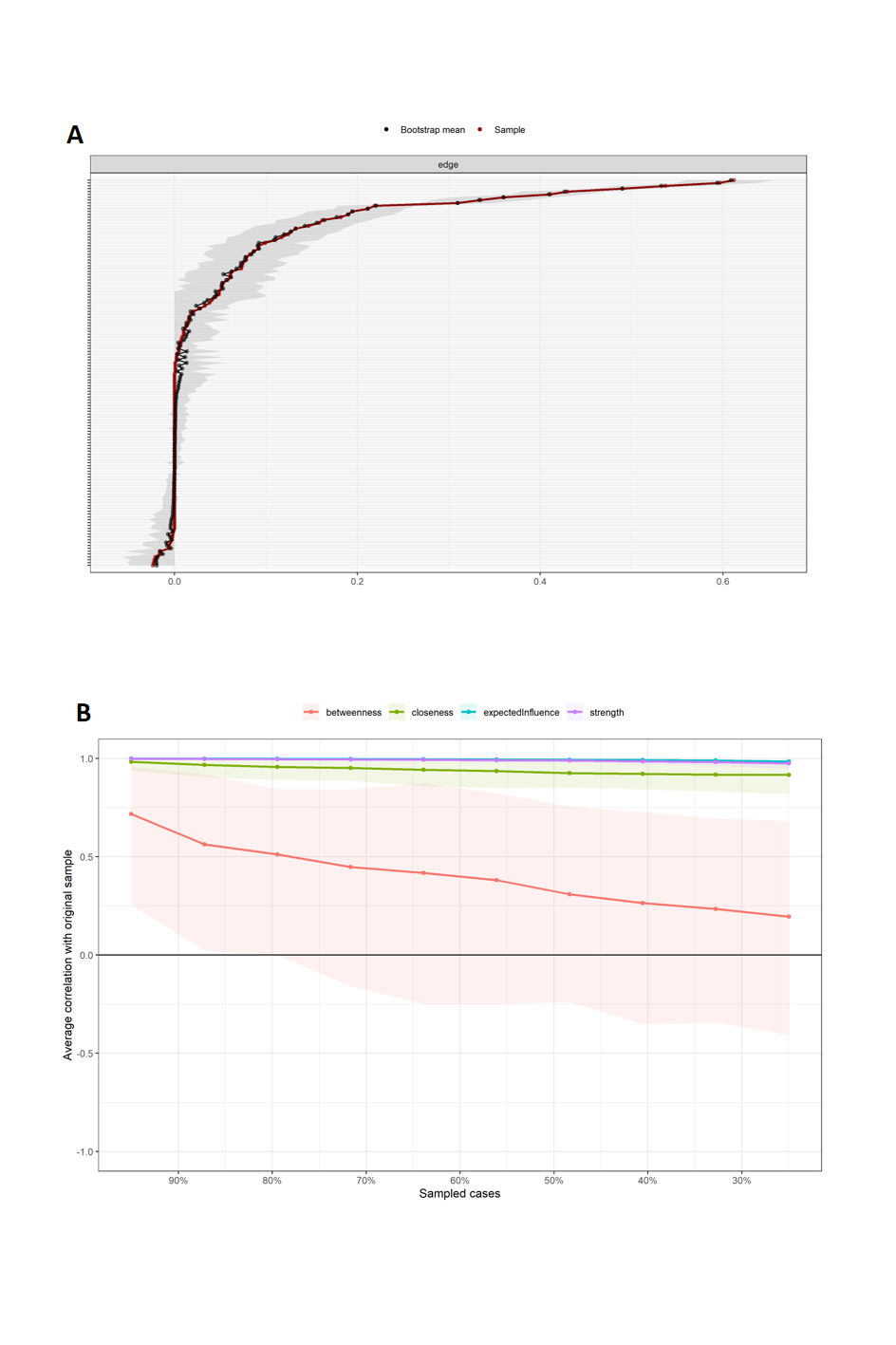


**Note:** Black lines represent the bootstrapped mean edge weights, while red lines represent the edge weights in the study sample. The gray area represents the bootstrap 95% confidence interval.

**Supplemental Figure 8** The accuracy (A) and stability indices (B) of network 2


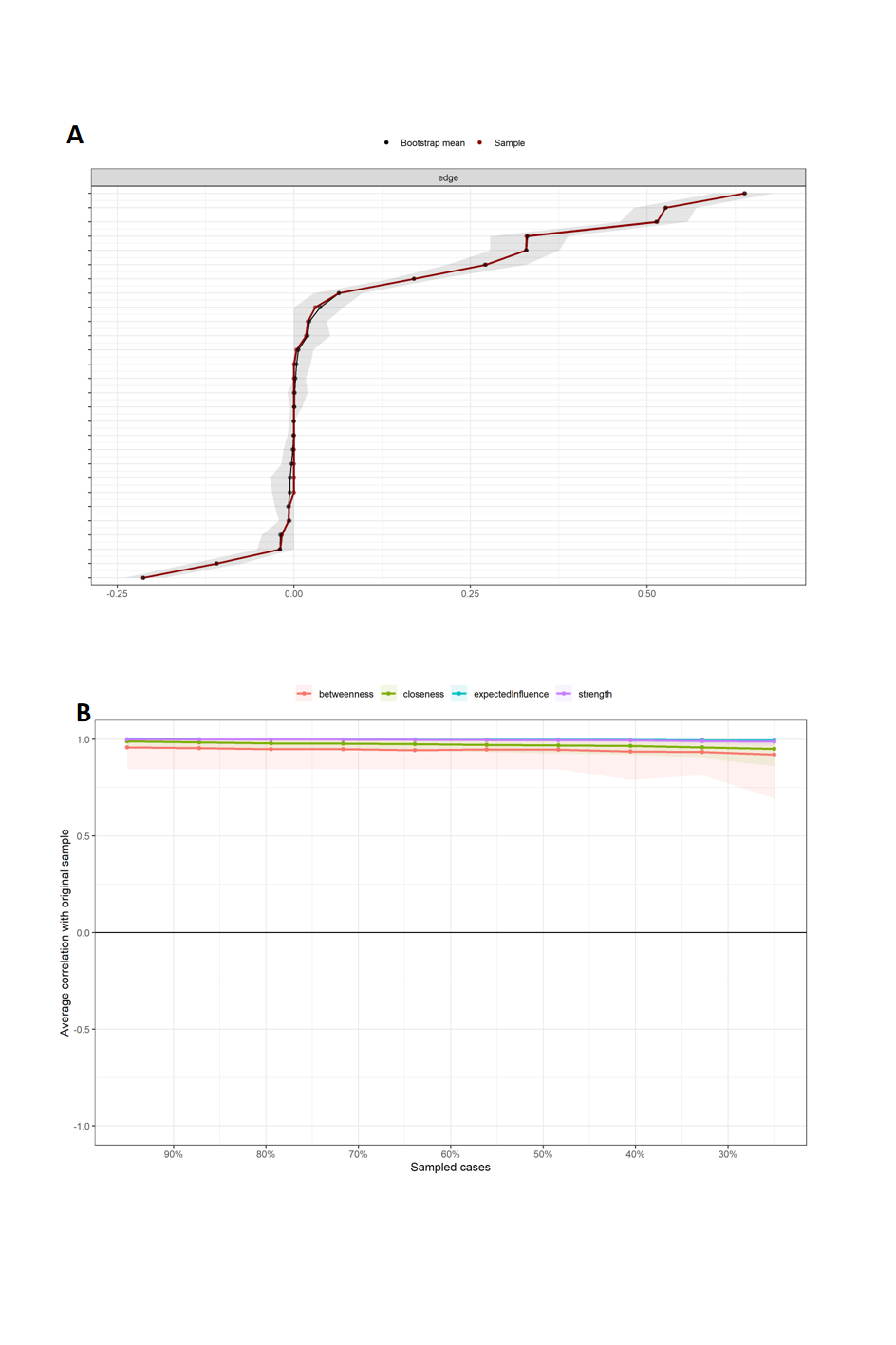


**Note:** Black lines represent the bootstrapped mean edge weights, while red lines represent the edge weights in the study sample. The gray area represents the bootstrap 95% confidence interval.

**Supplemental Figure 9** The accuracy (A) and stability indices (B) of network 3


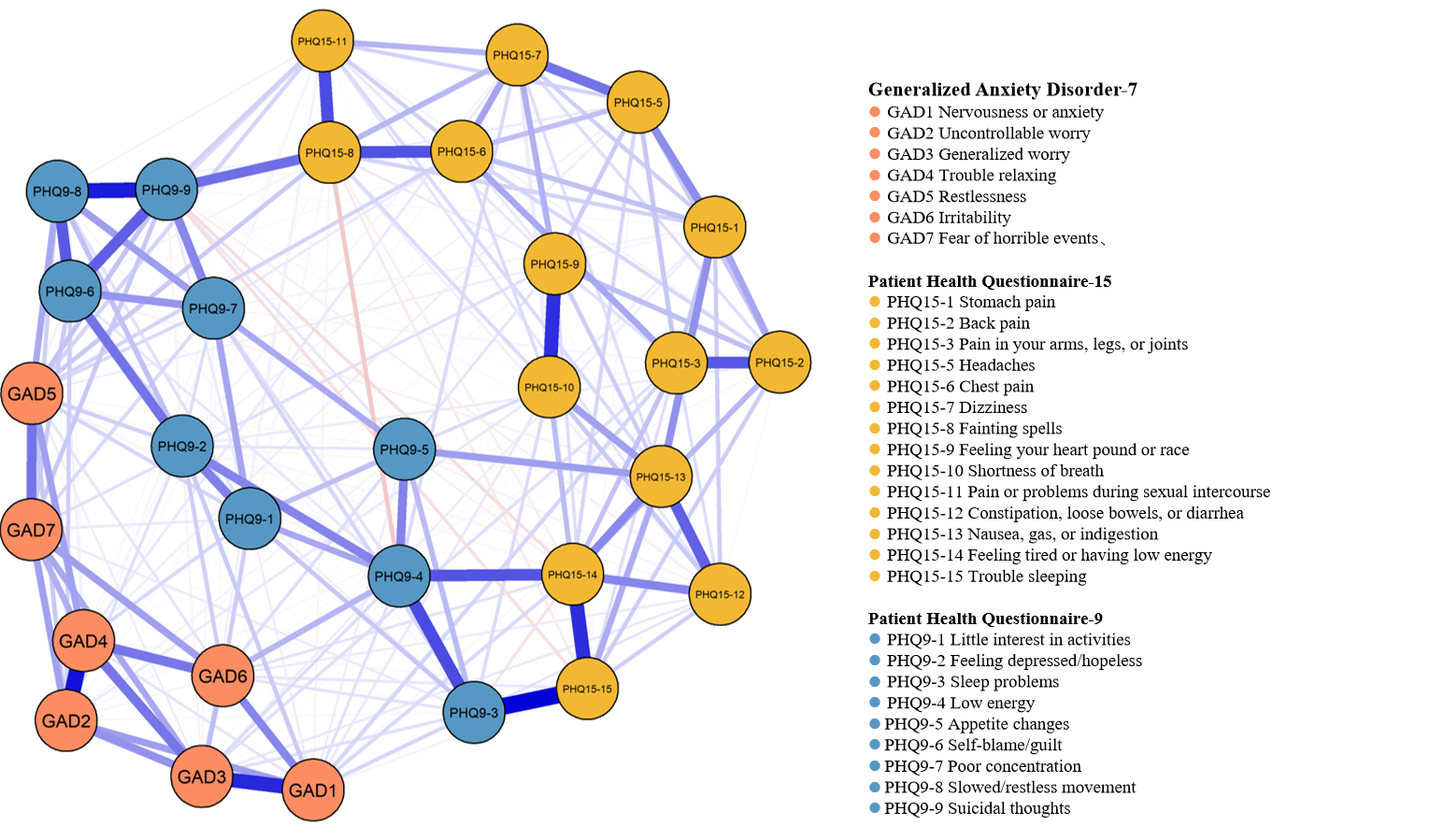


**Supplemental Figure 10** The network of mental health problems (Network 1) excluding the community of hopelessness


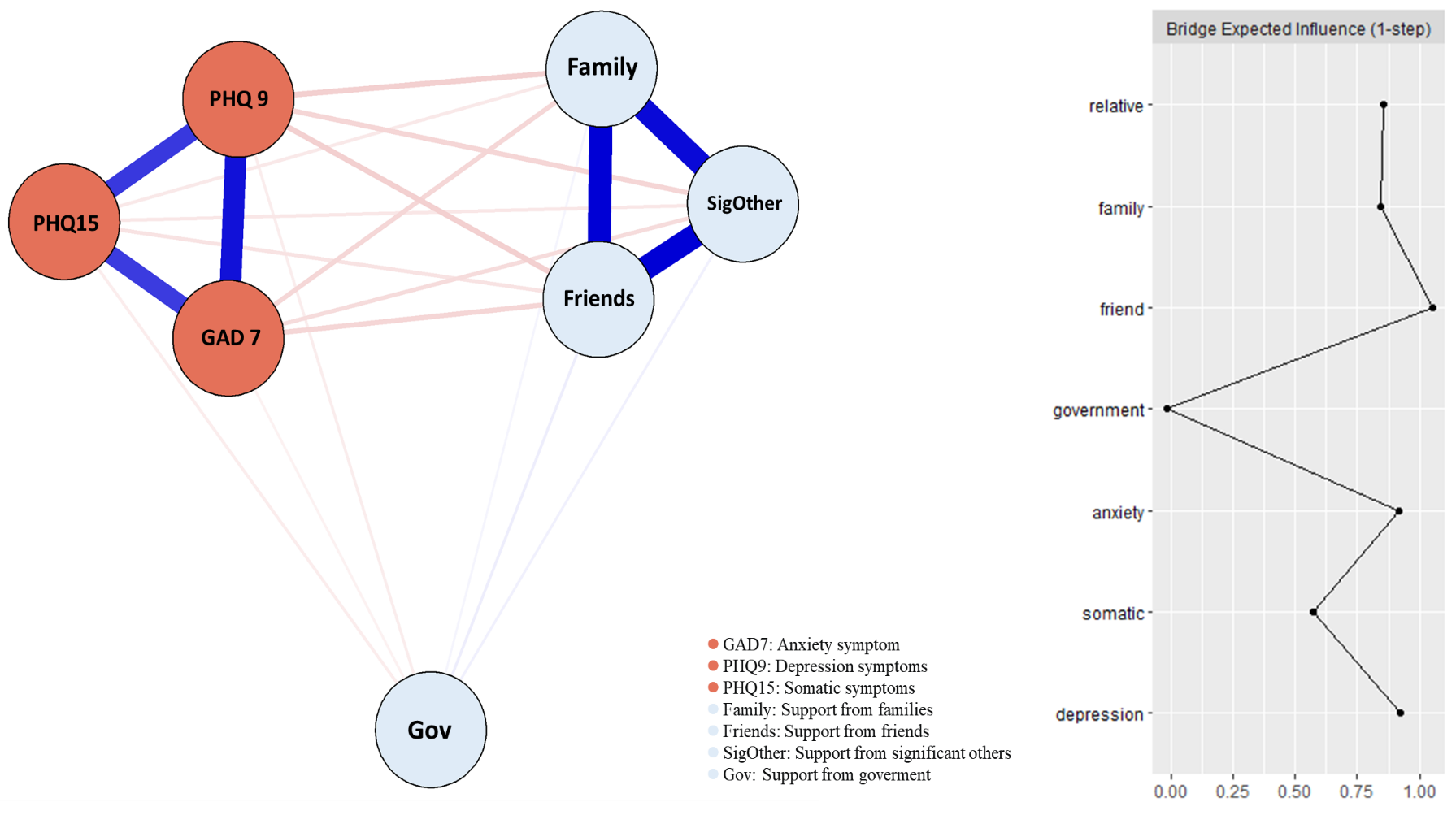


**Supplemental Figure 11** The network of mental health problems and perceived supports (Network 3) excluding the community of hopelessness


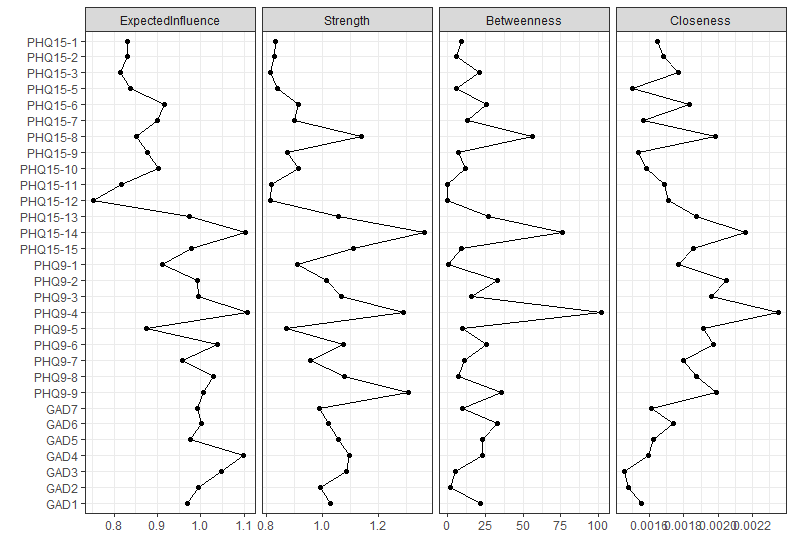


**Supplemental Figure 12** Centrality measures of network 1 excluding the community of hopelessness

**
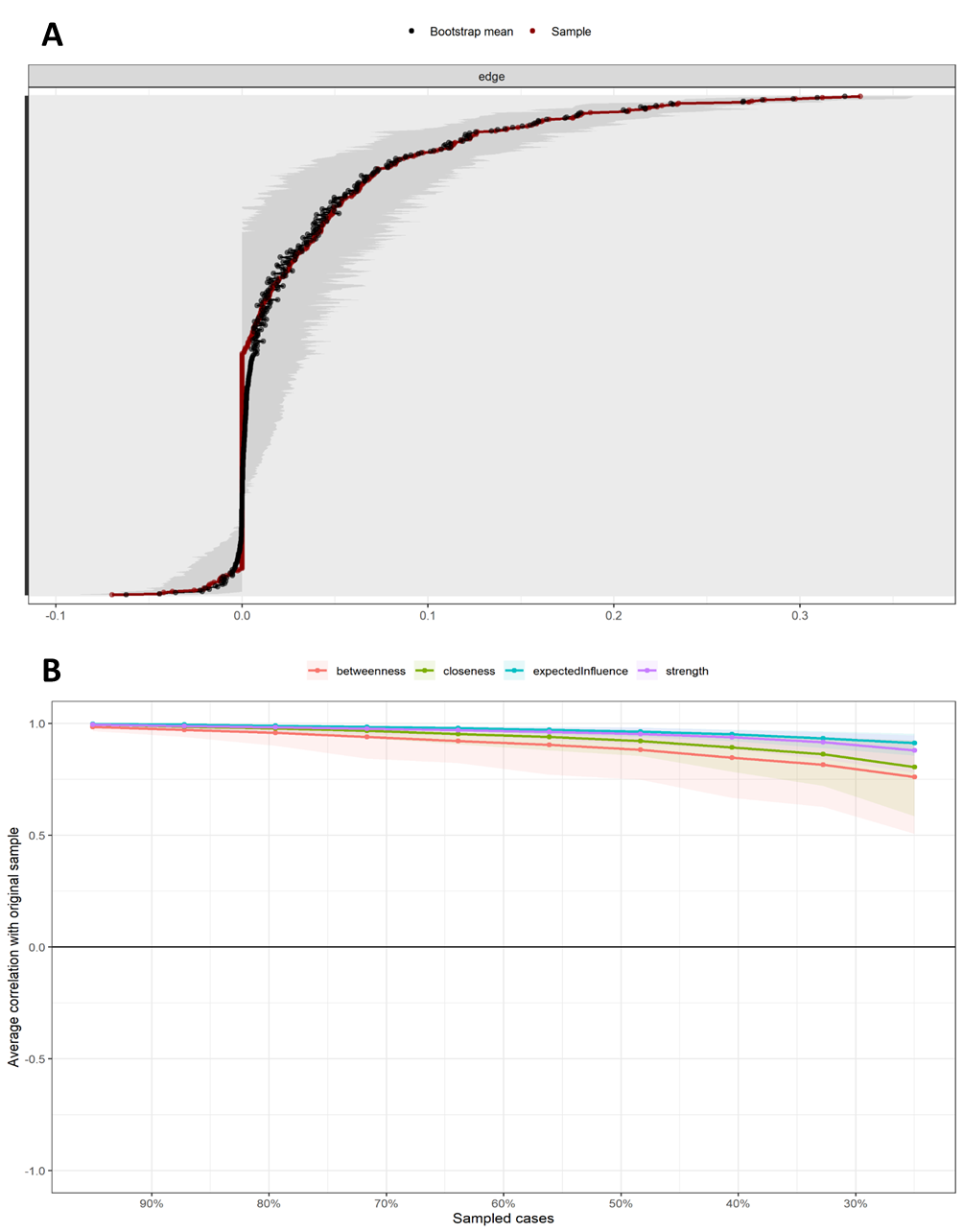
**

**Note:** Black lines represent the bootstrapped mean edge weights, while red lines represent the edge weights in the study sample. The gray area represents the bootstrap 95% confidence interval

**Supplemental Figure 13** The accuracy (A) and stability indices (B) of Network 1 excluding the community of hopelessnes


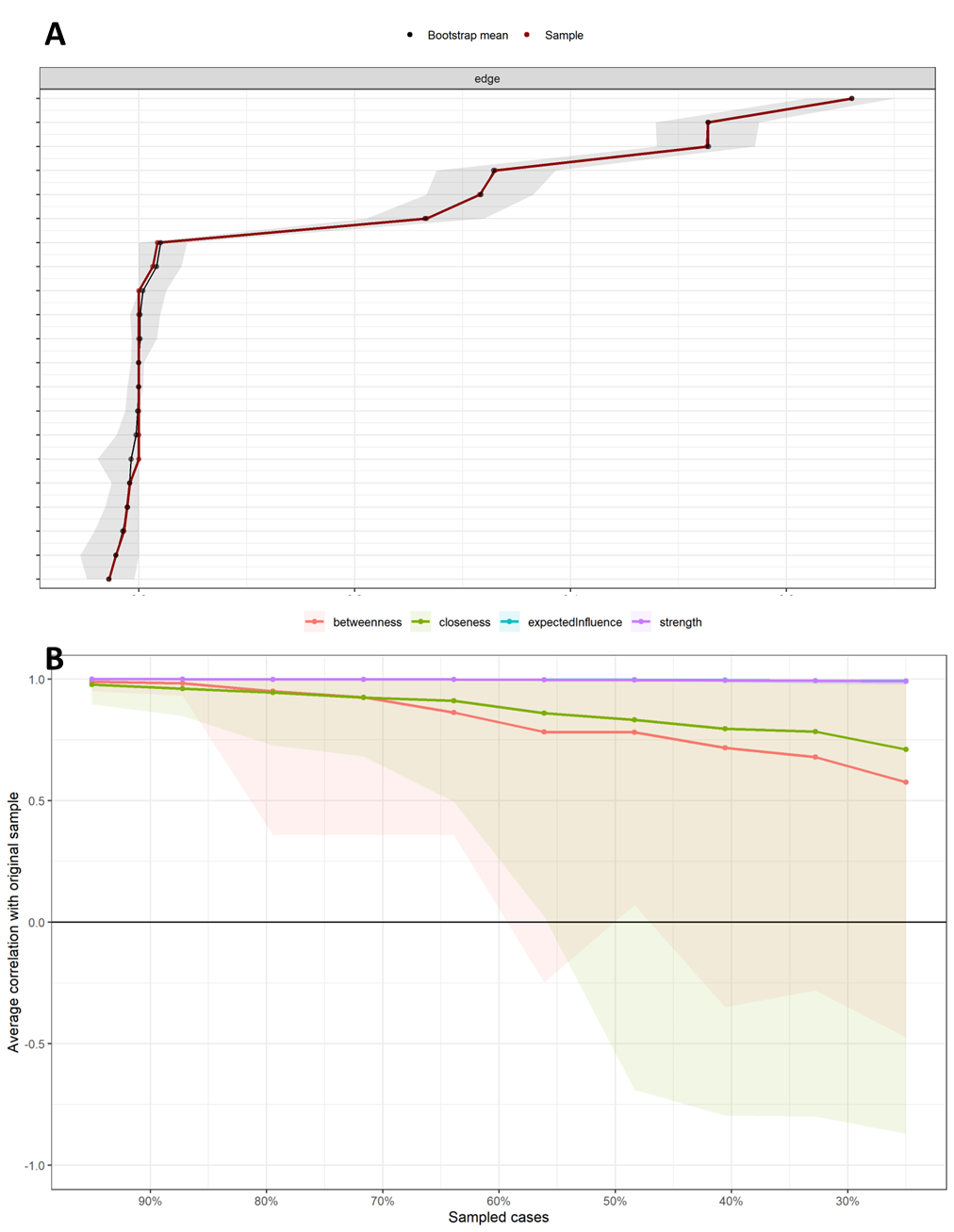


**Note:** Black lines represent the bootstrapped mean edge weights, while red lines represent the edge weights in the study sample. The gray area represents the bootstrap 95% confidence interval

**Supplemental Figure 14** The accuracy (A) and stability indices (B) of Network3 excluding the community of hopelessness


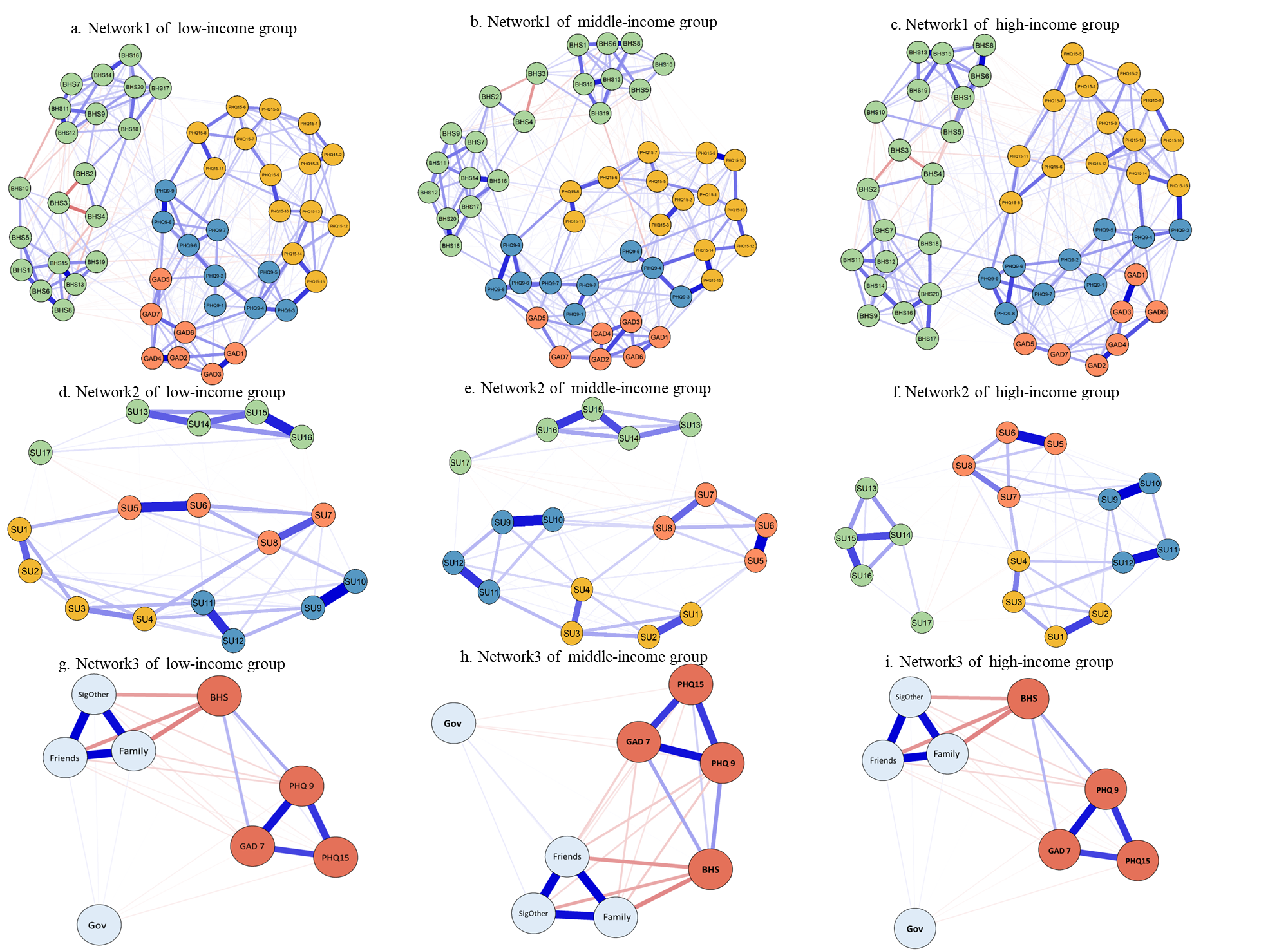
Note: The upper panel depicts the mental health network, while the lower panel shows the social support network.

**Supplemental Figure 15** Networks of mental health and social support by income level


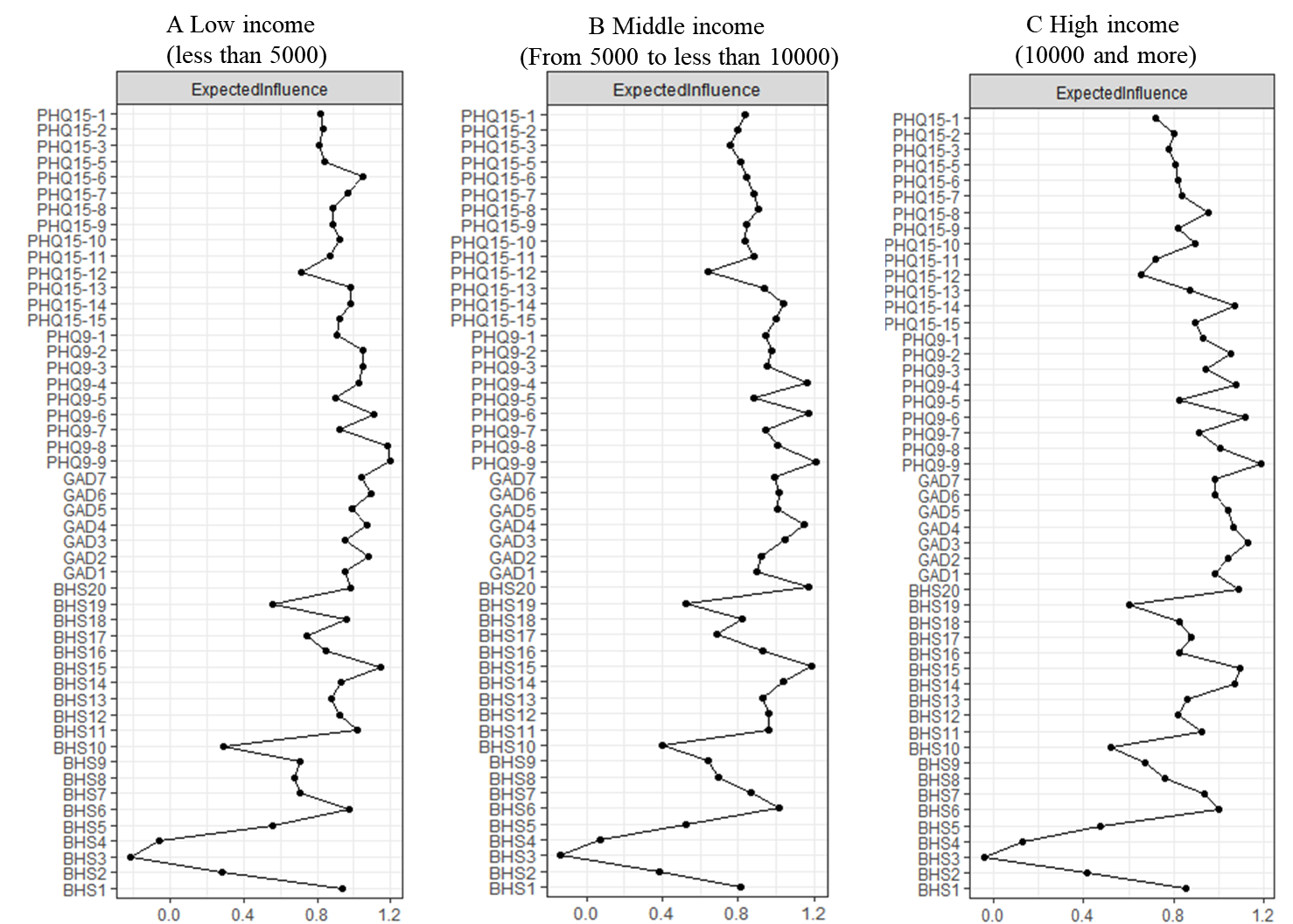


**Supplemental Figure 16** Centrality measures of networks of mental health by income level


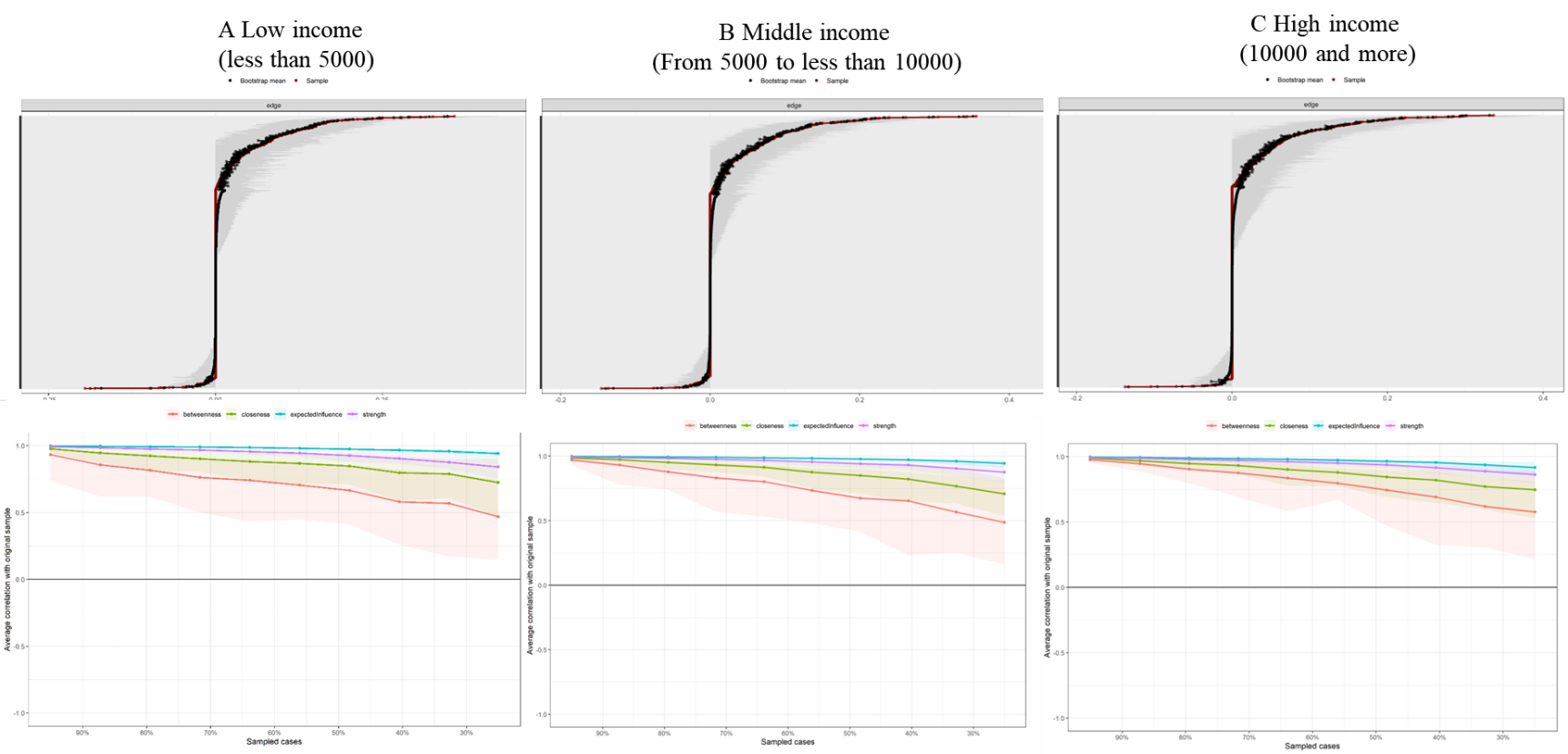


**Note:** Black lines represent the bootstrapped mean edge weights, while red lines represent the edge weights in the study sample. The gray area represents the bootstrap 95% confidence interval

**Supplemental Figure 17** The accuracy and stability indices of networks of mental health by income level


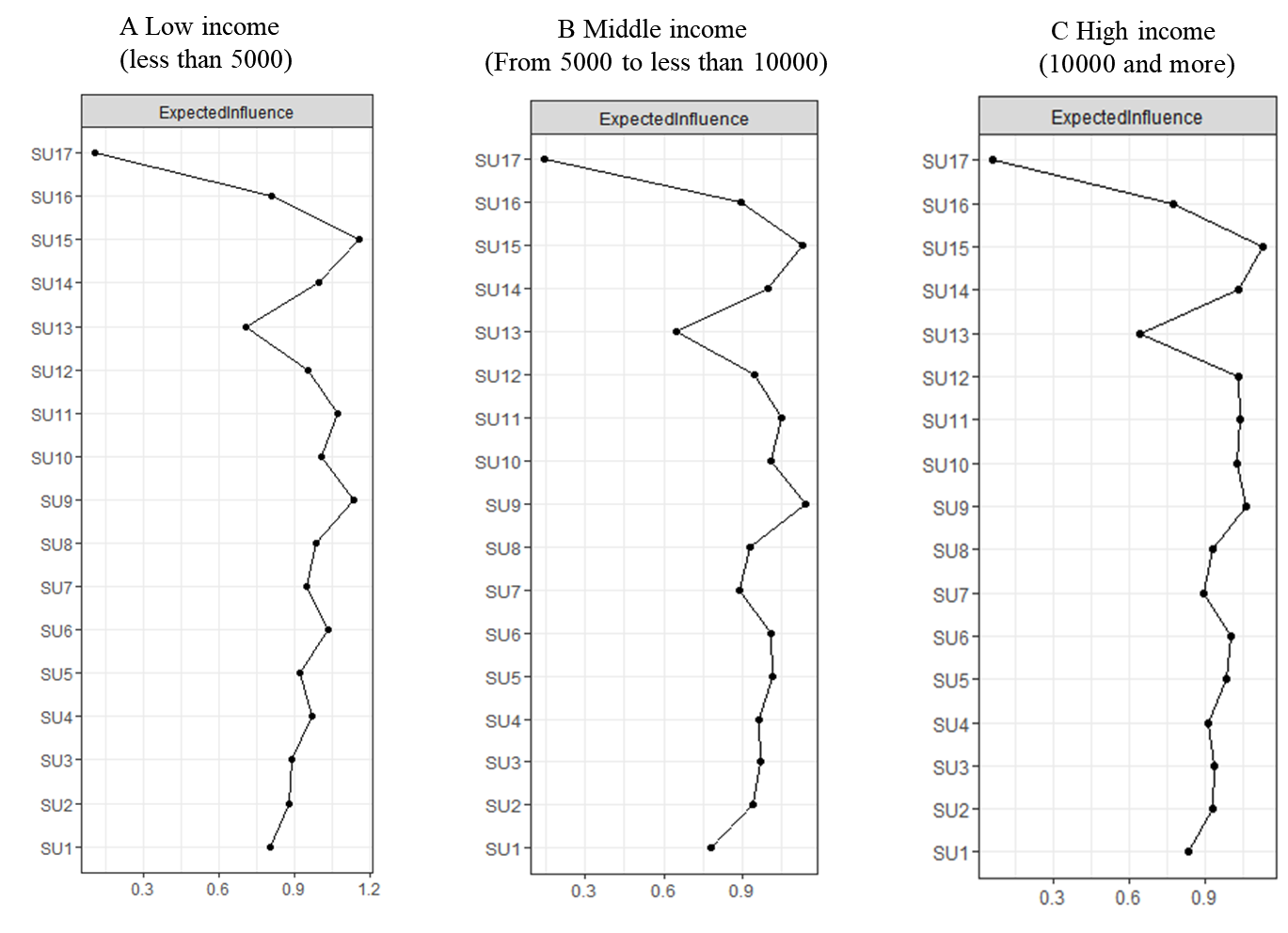


**Supplemental Figure 18** Centrality measures of networks of social support by income level


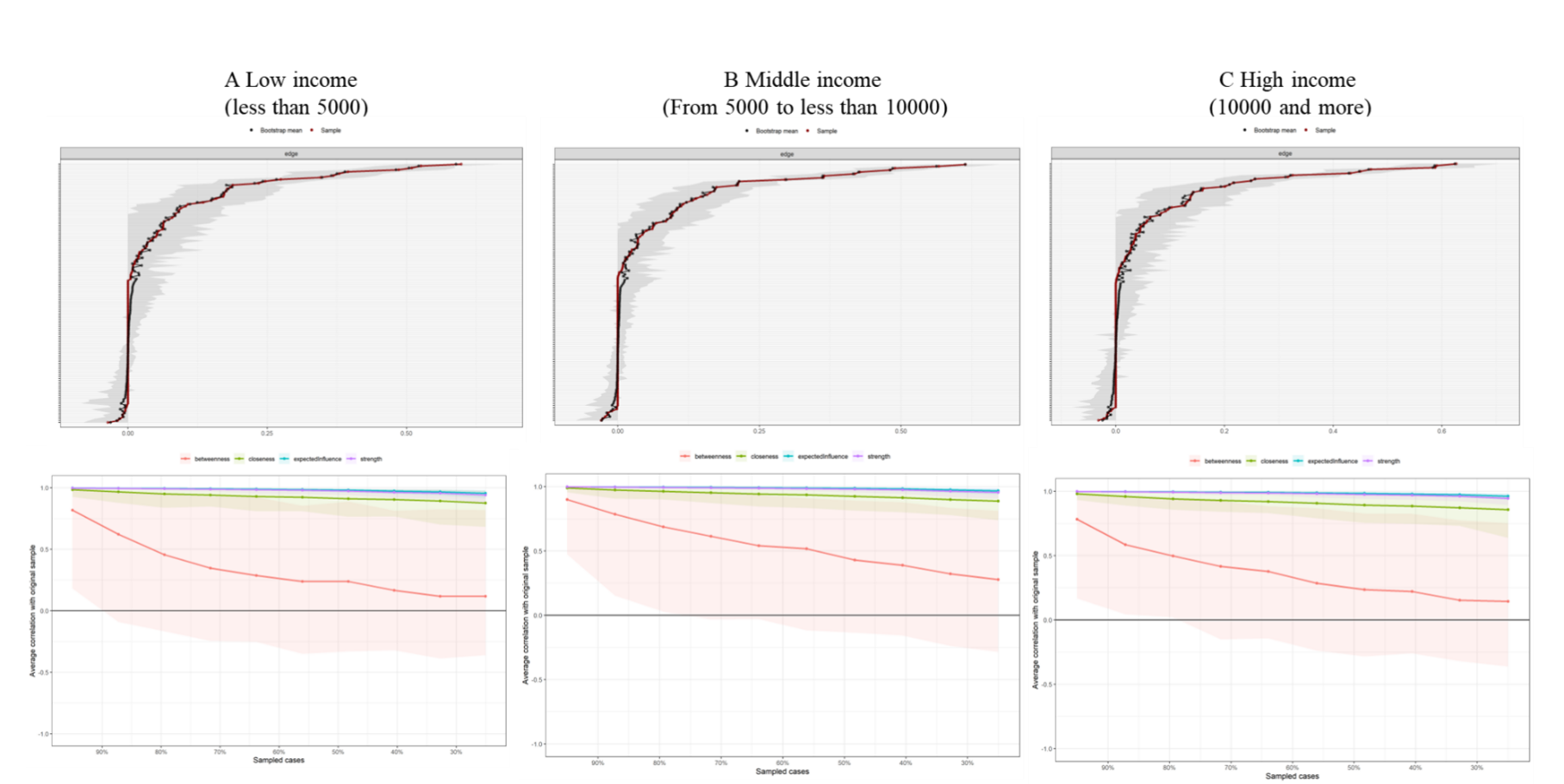


Note: Black lines represent the bootstrapped mean edge weights, while red lines represent the edge weights in the study sample. The gray area represents the bootstrap 95% confidence interval

**Supplemental Figure 19** The accuracy and stability indices of networks of social support by income level

**
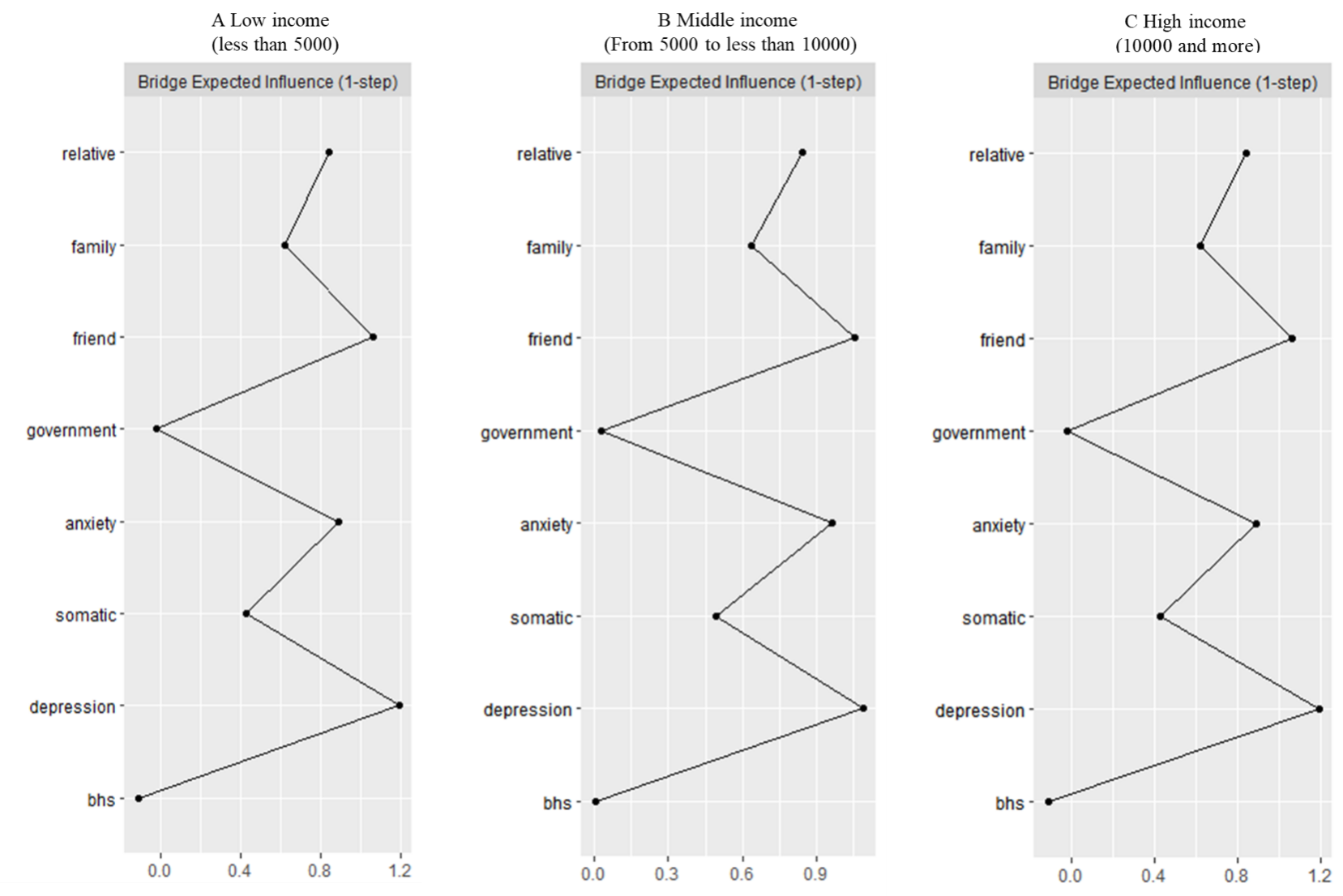
**

**Supplemental Figure 20** Centrality measures of network of mental health problems and perceived supports by income level

**
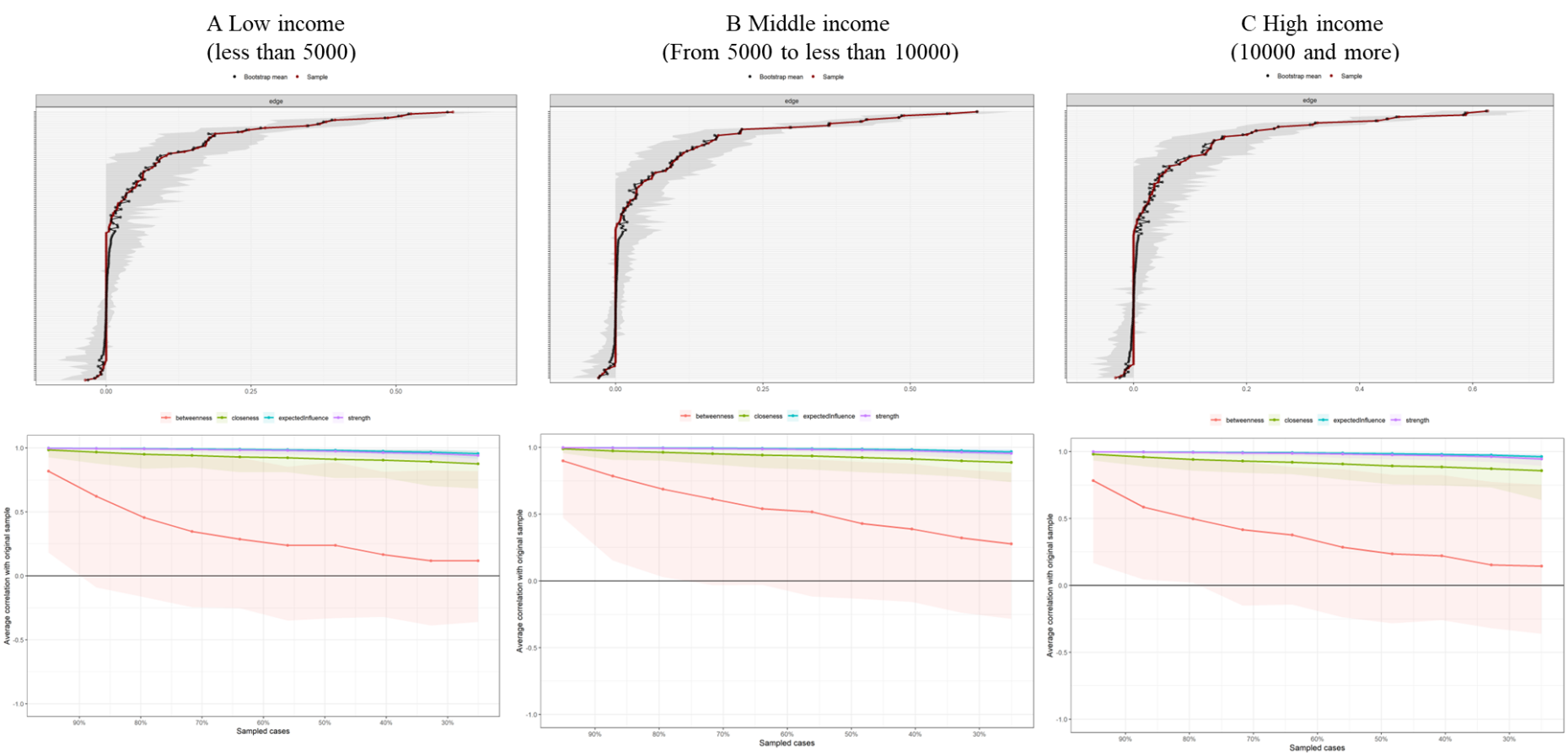
**

**Supplemental Figure 21** The accuracy and stability indices of networks of mental health problems and perceived supports by income level

**Supplemental Table1 Fertility Support Policies in detail**

| **Dimensions** | **Policy Item** |
| --- | --- |
| **Health Management** | Free prenatal physical examination, free newborn vaccinations, childbirth assistance, free newborn disease screening, postpartum rehabilitation, child health insurance |
| **Time Support** | Maternity leave and paternity leave, perinatal maternity leave, parental leave, family care leave |
| **Financial Assistance** | Maternity insurance, maternity allowance, income subsidies for mothers and spouses, tax exemption for child welfare income, family tax exemption, housing subsidies |
| **Service Provision** | Childcare services, parenting guidance services, after-school temporary care services |
| **Employment Protection** | Wage protection for pregnant women and mothers, employment guidance, job stability protection |

**Supplemental Table 2** Basic characteristic of the included participants

| **Characteristics** | **Overall (N=2989)**  **(n, %)** | **Antenatal (N=2090)**  **(n, %)**  **(n,%)** | **Postpartum (N=899)**  **(n, %)** | **t/X^2^**  **(*p*)** |
| --- | --- | --- | --- | --- |
| **Mean age** (years, SD) | 30.6 (5.33) | 29.8 (4.70) | 32.6 (6.14) | -12.15 (<0.001) |
| **Ethnicity** |  |  |  | 0.25 (0.62) |
| Han | 2930 (98.0%) | 2047 (97.9%) | 883 (98.2%) |  |
| Others ^ | 59 (2.0%) | 43 (2.1%) | 16 (1.8%) |  |
| **Number of children*** |  |  |  | 15.03 (<0.001) |
| One | 1975 (66.1%) | 1427 (68.3%) | 548 (61.0%) |  |
| Two or more | 1014 (33.9%) | 663 (31.7%) | 351 (39.0%) |  |
| **Monthly household income (CNY)** |  |  |  | 0.08 (0.96) |
| Less than 5000 | 822 (27.5%) | 578 (27.7%) | 244 (27.1%) |  |
| From 5000 to less than 10000 | 1302 (43.6%) | 908 (43.4%) | 394 (43.8%) |  |
| 10000 and more | 865 (28.9%) | 604 (28.9%) | 261 (29.0%) |  |
| **Educational level** |  |  |  | 4.67 (0.10) |
| Primary school or below | 81 (2.7%) | 56 (2.7%) | 25 (2.8%) |  |
| Middle school | 510 (17.1%) | 377 (18.0%) | 133 (14.8%) |  |
| College or above | 2398 (80.2%) | 1657 (79.3%) | 741 (82.4%) |  |
| **Employment status** |  |  |  | 12.26 (<0.001) |
| Unemployed | 624 (20.9%) | 472 (22.6%) | 152 (16.9%) |  |
| Other | 2365 (79.1%) | 1618 (77.4%) | 747 (83.1%) |  |
| **Partner’s educational level** |  |  |  | 0.23 (0.89) |
| Primary school or below | 80 (2.7%) | 54 (2.6%) | 26 (2.9%) |  |
| Middle school | 604 (20.2%) | 422 (20.2%) | 182 (20.2%) |  |
| College or above | 2305 (77.1%) | 1614 (77.2%) | 691 (76.9%) |  |
| **Partner’s employment status** |  |  |  | 0.003 (0.95) |
| Unemployed | 154 (5.2%) | 108 (5.2%) | 46 (5.1%) |  |
| Other | 2835 (94.8%) | 1982 (94.8%) | 853 (94.9%) |  |

^: other ethnic group includes Tibetan, Hui, etc.

Note: CNY, Chinese Yuan; SD, Standard deviation

* For participants in the prenatal period, including the soon-to-be-born baby

**Supplemental Table 3** The prevalence of depressive symptoms and anxiety symptoms

| **Symptoms** | **Overall (N=2989)**  **(n, %)** | **Antenatal (N=2090)**  **(n, %)**  **(n,%)** | **Postpartum (N=899)**  **(n, %)** | **t/X^2^**  **(*p*)** |
| --- | --- | --- | --- | --- |
| **Depressive symptom** |  |  |  | 2.110 (0.146) |
| No | 2768 (92.61) | 1945 (93.06) | 823 (91.55) |  |
| Probable depression | 221 (7.39) | 145 (6.94) | 76 (8.45) |  |
| **Anxiety symptom** |  |  |  | 13.933 (<0.001) |
| No | 2824 (94.48) | 1996 (95.50) | 828 (92.10) |  |
| Probable anxiety | 165 (5.52) | 94 (4.50) | 71 (7.90) |  |

**Supplemental Table 4** Edge weight of Network 1

|  | **1** | **2** | **3** | **4** | **5** | **6** | **7** | **8** | **9** | **10** | **11** | **12** | **13** | **14** | **15** | **16** | **17** | **18** | **19** | **20** | **21** | **22** | **23** | **24** | **25** | **26** | **27** | **28** | **29** | **30** | **31** | **32** | **33** | **34** | **35** | **36** | **37** | **38** | **39** | **40** | **41** | **42** | **43** | **44** | **45** | **46** | **47** | **48** | **49** | **50** |
| --- | --- | --- | --- | --- | --- | --- | --- | --- | --- | --- | --- | --- | --- | --- | --- | --- | --- | --- | --- | --- | --- | --- | --- | --- | --- | --- | --- | --- | --- | --- | --- | --- | --- | --- | --- | --- | --- | --- | --- | --- | --- | --- | --- | --- | --- | --- | --- | --- | --- | --- |
| **1: PHQ15-1** |  |  |  |  |  |  |  |  |  |  |  |  |  |  |  |  |  |  |  |  |  |  |  |  |  |  |  |  |  |  |  |  |  |  |  |  |  |  |  |  |  |  |  |  |  |  |  |  |  |  |
| **2: PHQ15-2** | 0.11 |  |  |  |  |  |  |  |  |  |  |  |  |  |  |  |  |  |  |  |  |  |  |  |  |  |  |  |  |  |  |  |  |  |  |  |  |  |  |  |  |  |  |  |  |  |  |  |  |  |
| **3: PHQ15-3** | 0.07 | 0.22 |  |  |  |  |  |  |  |  |  |  |  |  |  |  |  |  |  |  |  |  |  |  |  |  |  |  |  |  |  |  |  |  |  |  |  |  |  |  |  |  |  |  |  |  |  |  |  |  |
| **4: PHQ15-5** | 0.15 | 0.07 | 0.07 |  |  |  |  |  |  |  |  |  |  |  |  |  |  |  |  |  |  |  |  |  |  |  |  |  |  |  |  |  |  |  |  |  |  |  |  |  |  |  |  |  |  |  |  |  |  |  |
| **5: PHQ15-6** | 0.08 | 0.05 | 0.11 | 0.08 |  |  |  |  |  |  |  |  |  |  |  |  |  |  |  |  |  |  |  |  |  |  |  |  |  |  |  |  |  |  |  |  |  |  |  |  |  |  |  |  |  |  |  |  |  |  |
| **6: PHQ15-7** | 0.06 | 0.04 | 0 | 0.18 | 0.12 |  |  |  |  |  |  |  |  |  |  |  |  |  |  |  |  |  |  |  |  |  |  |  |  |  |  |  |  |  |  |  |  |  |  |  |  |  |  |  |  |  |  |  |  |  |
| **7: PHQ15-8** | 0.06 | 0 | 0 | 0 | 0.22 | 0.09 |  |  |  |  |  |  |  |  |  |  |  |  |  |  |  |  |  |  |  |  |  |  |  |  |  |  |  |  |  |  |  |  |  |  |  |  |  |  |  |  |  |  |  |  |
| **8: PHQ15-9** | 0.01 | 0.08 | 0.04 | 0.07 | 0.03 | 0.10 | 0.02 |  |  |  |  |  |  |  |  |  |  |  |  |  |  |  |  |  |  |  |  |  |  |  |  |  |  |  |  |  |  |  |  |  |  |  |  |  |  |  |  |  |  |  |
| **9: PHQ15-10** | 0.02 | 0.01 | 0.01 | 0 | 0.05 | 0.03 | 0.04 | 0.27 |  |  |  |  |  |  |  |  |  |  |  |  |  |  |  |  |  |  |  |  |  |  |  |  |  |  |  |  |  |  |  |  |  |  |  |  |  |  |  |  |  |  |
| **10: PHQ15-11** | 0.01 | 0.02 | 0 | 0.06 | 0.06 | 0.10 | 0.22 | 0.03 | 0 |  |  |  |  |  |  |  |  |  |  |  |  |  |  |  |  |  |  |  |  |  |  |  |  |  |  |  |  |  |  |  |  |  |  |  |  |  |  |  |  |  |
| **11: PHQ15-12** | 0.06 | 0.01 | 0.04 | 0 | 0.01 | 0 | 0 | 0 | 0.07 | 0.04 |  |  |  |  |  |  |  |  |  |  |  |  |  |  |  |  |  |  |  |  |  |  |  |  |  |  |  |  |  |  |  |  |  |  |  |  |  |  |  |  |
| **12: PHQ15-13** | 0.14 | 0 | 0 | 0 | 0 | 0.04 | 0 | 0.06 | 0.12 | 0.04 | 0.20 |  |  |  |  |  |  |  |  |  |  |  |  |  |  |  |  |  |  |  |  |  |  |  |  |  |  |  |  |  |  |  |  |  |  |  |  |  |  |  |
| **13: PHQ15-14** | 0 | 0.09 | 0.08 | 0.05 | 0 | 0.02 | 0 | 0 | 0.08 | 0 | 0.15 | 0.15 |  |  |  |  |  |  |  |  |  |  |  |  |  |  |  |  |  |  |  |  |  |  |  |  |  |  |  |  |  |  |  |  |  |  |  |  |  |  |
| **14: PHQ15-15** | 0 | 0.06 | 0.04 | 0.01 | 0 | 0 | -0.01 | 0.04 | 0.05 | 0 | 0.06 | 0.10 | 0.26 |  |  |  |  |  |  |  |  |  |  |  |  |  |  |  |  |  |  |  |  |  |  |  |  |  |  |  |  |  |  |  |  |  |  |  |  |  |
| **15: GAD7** | 0 | 0 | 0 | 0 | 0 | 0 | 0 | 0.04 | 0 | 0 | 0 | 0 | 0 | 0 |  |  |  |  |  |  |  |  |  |  |  |  |  |  |  |  |  |  |  |  |  |  |  |  |  |  |  |  |  |  |  |  |  |  |  |  |
| **16: GAD6** | 0 | 0.01 | 0.01 | 0 | 0 | 0 | 0 | 0 | 0 | 0 | 0.02 | 0 | 0.03 | 0.04 | 0.12 |  |  |  |  |  |  |  |  |  |  |  |  |  |  |  |  |  |  |  |  |  |  |  |  |  |  |  |  |  |  |  |  |  |  |  |
| **17: GAD5** | 0 | 0 | 0 | 0 | 0.05 | 0 | 0.05 | 0.02 | 0 | 0.06 | 0 | 0 | 0 | 0 | 0.18 | 0.03 |  |  |  |  |  |  |  |  |  |  |  |  |  |  |  |  |  |  |  |  |  |  |  |  |  |  |  |  |  |  |  |  |  |  |
| **18: GAD4** | 0 | 0 | 0.01 | 0.02 | 0 | 0.03 | 0 | 0 | 0 | 0.01 | 0 | 0 | 0 | 0 | 0.11 | 0.17 | 0.12 |  |  |  |  |  |  |  |  |  |  |  |  |  |  |  |  |  |  |  |  |  |  |  |  |  |  |  |  |  |  |  |  |  |
| **19: GAD3** | 0.02 | 0 | 0.04 | 0.01 | 0 | 0 | 0 | 0 | 0 | 0.02 | 0.03 | 0 | 0 | 0 | 0.11 | 0.09 | 0.02 | 0.18 |  |  |  |  |  |  |  |  |  |  |  |  |  |  |  |  |  |  |  |  |  |  |  |  |  |  |  |  |  |  |  |  |
| **20: GAD2** | 0 | 0.01 | 0 | 0 | 0.01 | 0 | 0 | 0 | 0.03 | 0.02 | 0 | 0 | 0 | 0 | 0.12 | 0.02 | 0.07 | 0.31 | 0.14 |  |  |  |  |  |  |  |  |  |  |  |  |  |  |  |  |  |  |  |  |  |  |  |  |  |  |  |  |  |  |  |
| **21: GAD1** | 0 | 0.01 | 0.05 | 0 | 0 | 0 | 0 | 0 | 0.03 | 0 | 0.03 | 0 | 0.04 | 0.02 | 0 | 0.15 | 0 | 0.04 | 0.28 | 0.12 |  |  |  |  |  |  |  |  |  |  |  |  |  |  |  |  |  |  |  |  |  |  |  |  |  |  |  |  |  |  |
| **22: PHQ9-1** | 0 | 0 | 0 | 0 | 0 | 0 | 0 | 0.01 | 0 | 0 | 0 | 0 | 0 | 0 | 0.03 | 0.07 | 0.05 | 0.05 | 0.01 | 0 | 0.02 |  |  |  |  |  |  |  |  |  |  |  |  |  |  |  |  |  |  |  |  |  |  |  |  |  |  |  |  |  |
| **23: PHQ9-2** | 0 | 0.02 | 0 | 0.02 | 0 | 0.01 | 0 | 0 | 0 | 0 | 0 | 0 | 0 | 0 | 0 | 0.06 | 0.06 | 0.01 | 0.02 | 0.04 | 0.07 | 0.16 |  |  |  |  |  |  |  |  |  |  |  |  |  |  |  |  |  |  |  |  |  |  |  |  |  |  |  |  |
| **24: PHQ9-3** | 0 | 0 | 0 | 0 | 0 | 0 | 0 | 0 | 0.04 | 0 | 0 | 0 | 0 | 0.31 | 0.03 | 0.04 | 0 | 0.01 | 0.04 | 0.01 | 0.05 | 0.07 | 0.02 |  |  |  |  |  |  |  |  |  |  |  |  |  |  |  |  |  |  |  |  |  |  |  |  |  |  |  |
| **25: PHQ9-4** | 0 | 0.01 | 0 | 0 | 0 | 0.05 | -0.05 | 0 | 0 | 0 | 0 | 0 | 0.20 | 0 | 0 | 0.10 | 0 | 0 | 0.04 | 0 | 0.04 | 0.11 | 0.16 | 0.22 |  |  |  |  |  |  |  |  |  |  |  |  |  |  |  |  |  |  |  |  |  |  |  |  |  |  |
| **26: PHQ9-5** | 0.01 | 0 | 0 | 0 | 0 | 0 | 0 | 0.04 | 0.05 | 0 | 0.01 | 0.11 | 0 | 0 | 0 | 0.05 | 0.01 | 0 | 0 | 0.02 | 0.02 | 0.08 | 0.03 | 0.09 | 0.16 |  |  |  |  |  |  |  |  |  |  |  |  |  |  |  |  |  |  |  |  |  |  |  |  |  |
| **27: PHQ9-6** | 0 | 0 | 0 | 0.01 | 0 | 0.01 | 0 | 0 | 0 | 0.04 | 0 | 0 | 0 | 0 | 0.07 | 0 | 0.01 | 0.01 | 0 | 0.06 | 0 | 0.05 | 0.18 | 0 | 0.02 | 0.01 |  |  |  |  |  |  |  |  |  |  |  |  |  |  |  |  |  |  |  |  |  |  |  |  |
| **28: PHQ9-7** | 0.01 | 0 | 0.01 | 0 | 0.03 | 0 | 0 | 0 | 0 | 0 | 0 | 0 | 0 | 0 | 0.05 | 0.01 | 0.05 | 0 | 0.01 | 0 | 0 | 0.12 | 0.05 | 0.05 | 0.03 | 0.11 | 0.14 |  |  |  |  |  |  |  |  |  |  |  |  |  |  |  |  |  |  |  |  |  |  |  |
| **29: PHQ9-8** | 0 | 0 | 0 | 0 | 0 | 0 | 0 | 0 | 0 | 0.02 | 0 | 0 | 0 | 0 | 0.06 | 0 | 0.11 | 0.01 | 0 | 0.01 | 0 | 0.04 | 0.08 | 0.01 | 0 | 0.04 | 0.21 | 0.13 |  |  |  |  |  |  |  |  |  |  |  |  |  |  |  |  |  |  |  |  |  |  |
| **30: PHQ9-9** | 0 | 0 | 0 | 0.02 | 0 | 0 | 0.16 | 0 | 0 | 0.05 | 0 | 0 | -0.03 | -0.02 | 0.06 | 0 | 0.07 | 0 | 0 | 0 | 0 | 0.02 | 0 | 0 | 0 | 0.02 | 0.18 | 0.15 | 0.28 |  |  |  |  |  |  |  |  |  |  |  |  |  |  |  |  |  |  |  |  |  |
| **31: BHS1** | 0 | 0 | 0 | 0 | 0 | 0 | 0 | 0 | 0 | 0.02 | 0 | 0 | 0 | 0 | 0 | 0 | 0 | 0 | 0 | 0 | -0.01 | 0 | 0 | 0 | 0 | 0 | 0.05 | 0 | 0 | 0.01 |  |  |  |  |  |  |  |  |  |  |  |  |  |  |  |  |  |  |  |  |
| **32: BHS2** | 0 | 0 | 0 | 0 | 0 | 0 | 0 | 0 | 0 | 0 | 0 | 0 | 0 | 0 | 0 | 0 | 0 | 0 | 0 | 0 | 0 | 0 | 0 | 0 | 0 | 0 | 0.04 | 0 | 0 | 0 | 0 |  |  |  |  |  |  |  |  |  |  |  |  |  |  |  |  |  |  |  |
| **33: BHS3** | 0 | 0 | -0.01 | 0 | 0 | 0 | 0 | 0 | 0 | 0 | -0.04 | 0 | -0.02 | 0 | 0 | 0 | 0 | 0 | 0 | 0 | 0 | 0 | 0 | 0 | 0 | 0 | 0 | -0.02 | 0 | 0 | 0.07 | -0.15 |  |  |  |  |  |  |  |  |  |  |  |  |  |  |  |  |  |  |
| **34: BHS4** | 0 | 0 | 0 | 0 | 0 | 0 | -0.01 | 0 | 0 | 0 | 0 | 0 | 0.02 | 0 | 0 | 0.01 | 0 | 0 | 0 | 0 | 0 | 0.02 | 0.02 | 0.02 | 0 | 0 | 0 | 0 | 0 | 0 | -0.04 | 0.15 | -0.16 |  |  |  |  |  |  |  |  |  |  |  |  |  |  |  |  |  |
| **35: BHS5** | 0 | 0 | 0 | 0.01 | 0 | 0 | -0.02 | 0 | -0.03 | 0 | 0 | -0.02 | 0.04 | 0.01 | 0 | 0.02 | 0 | 0.03 | 0 | 0 | 0 | 0.02 | 0 | 0 | 0.03 | 0 | 0 | 0 | 0 | 0 | 0.12 | 0 | 0 | 0.04 |  |  |  |  |  |  |  |  |  |  |  |  |  |  |  |  |
| **36: BHS6** | 0 | 0 | -0.01 | 0 | 0 | 0 | 0.05 | 0 | 0 | 0 | -0.01 | 0 | -0.03 | 0 | 0 | 0 | 0 | 0 | 0 | 0 | 0 | 0 | 0 | 0 | -0.01 | 0 | 0 | 0 | 0.02 | 0.03 | 0.21 | -0.02 | 0.05 | -0.08 | 0.16 |  |  |  |  |  |  |  |  |  |  |  |  |  |  |  |
| **37: BHS7** | 0 | 0 | 0 | 0.02 | 0.02 | 0 | 0.03 | 0 | 0 | 0.01 | -0.02 | 0 | -0.01 | 0 | 0 | 0 | 0.02 | 0 | 0 | 0 | 0 | 0 | 0 | 0 | 0 | 0 | 0 | 0 | 0 | 0.03 | 0.03 | 0.07 | -0.02 | 0.01 | -0.05 | 0 |  |  |  |  |  |  |  |  |  |  |  |  |  |  |
| **38: BHS8** | 0 | 0 | 0 | 0 | 0 | 0 | 0.02 | 0 | 0 | 0 | 0 | 0 | -0.02 | -0.02 | 0 | 0 | 0 | 0 | 0 | 0 | -0.02 | 0 | 0 | -0.01 | 0 | 0 | 0 | 0 | 0 | 0 | 0.12 | -0.02 | 0.01 | -0.07 | 0.05 | 0.33 | 0 |  |  |  |  |  |  |  |  |  |  |  |  |  |
| **39: BHS9** | 0 | 0 | 0 | 0 | 0 | 0 | 0 | 0 | 0 | 0 | 0 | 0 | 0 | -0.02 | 0 | 0 | 0 | 0 | 0 | 0 | 0 | 0 | 0 | 0 | -0.01 | 0 | 0 | 0 | 0 | 0 | 0 | 0.05 | 0 | 0.05 | -0.02 | 0 | 0.07 | 0 |  |  |  |  |  |  |  |  |  |  |  |  |
| **40: BHS10** | 0 | 0 | 0 | 0 | 0 | 0 | 0 | 0 | 0 | 0 | 0 | 0 | 0 | 0 | 0 | 0 | 0 | 0 | 0 | 0.01 | 0 | 0 | 0 | 0 | 0 | 0 | 0.01 | 0 | 0 | 0 | 0.02 | 0 | 0.07 | 0 | 0.12 | 0.05 | 0 | 0.04 | -0.05 |  |  |  |  |  |  |  |  |  |  |  |
| **41: BHS11** | 0 | 0 | 0 | 0 | 0 | 0 | 0 | 0 | -0.01 | 0 | 0 | 0 | -0.01 | 0 | 0.01 | 0 | 0.01 | 0 | 0 | 0 | 0 | 0 | 0 | 0 | 0 | 0 | 0 | 0 | 0 | 0.04 | 0 | 0.02 | -0.02 | 0 | 0 | 0 | 0.19 | 0 | 0.15 | -0.06 |  |  |  |  |  |  |  |  |  |  |
| **42: BHS12** | 0 | 0 | 0 | 0 | 0 | 0 | 0 | 0 | 0 | 0 | 0 | 0 | 0 | 0 | 0 | 0 | 0.02 | 0 | 0 | 0 | 0 | 0.04 | 0 | 0 | 0 | 0 | 0.03 | 0 | 0 | 0 | 0 | 0.06 | 0 | 0 | 0 | 0 | 0.07 | -0.01 | 0.15 | -0.01 | 0.22 |  |  |  |  |  |  |  |  |  |
| **43: BHS13** | 0 | 0 | 0 | 0 | 0 | 0 | 0 | 0 | 0 | 0.01 | -0.01 | -0.01 | 0 | 0 | 0 | 0 | 0 | 0 | 0 | 0 | -0.02 | 0 | 0 | 0 | -0.01 | 0 | 0 | 0 | 0 | 0.01 | 0.08 | 0 | 0.04 | 0 | 0 | 0.11 | 0 | 0.12 | 0 | 0.09 | 0 | 0 |  |  |  |  |  |  |  |  |
| **44: BHS14** | 0 | 0 | 0 | 0 | 0 | 0 | 0 | 0 | 0 | 0 | 0 | -0.02 | 0 | 0 | 0 | 0 | 0.01 | 0 | 0 | 0.01 | 0 | 0 | 0 | 0 | 0 | 0 | 0.01 | 0 | 0.01 | 0 | 0 | 0.06 | 0 | 0 | 0 | 0 | 0.11 | 0 | 0.03 | 0 | 0.17 | 0.12 | 0 |  |  |  |  |  |  |  |
| **45: BHS15** | 0 | 0 | 0 | 0 | 0 | 0 | 0 | 0 | 0 | 0 | 0 | 0 | 0 | 0 | 0 | 0 | 0 | 0 | 0 | 0 | 0 | 0 | 0 | 0 | 0 | -0.01 | 0 | 0 | 0 | 0.05 | 0.20 | 0 | 0 | 0 | 0.08 | 0.10 | 0 | 0.12 | 0 | 0.07 | 0 | 0 | 0.35 | 0 |  |  |  |  |  |  |
| **46: BHS16** | 0 | 0 | 0 | 0 | 0 | 0 | 0.03 | 0 | 0 | 0 | 0 | 0 | -0.01 | 0 | 0 | 0 | 0 | 0 | 0 | 0 | 0 | 0 | 0 | -0.02 | 0 | 0 | 0 | 0 | 0 | 0.04 | 0 | 0.01 | -0.02 | 0 | -0.03 | 0 | 0.07 | 0 | 0.07 | 0 | 0.09 | 0.04 | 0 | 0.26 | 0 |  |  |  |  |  |
| **47: BHS17** | 0 | 0 | 0 | 0 | 0.01 | 0 | 0.01 | 0 | 0 | 0 | -0.01 | 0 | -0.03 | 0 | 0 | 0 | 0.01 | 0 | 0 | 0 | 0 | 0 | 0 | 0 | 0 | 0 | 0 | 0 | 0.01 | 0.01 | 0 | 0.01 | -0.01 | 0 | -0.01 | 0 | 0.04 | 0 | 0.11 | -0.02 | 0.05 | 0.05 | 0 | 0.10 | 0 | 0.09 |  |  |  |  |
| **48: BHS18** | 0 | 0 | 0 | 0 | 0 | 0 | 0 | 0 | 0 | 0 | 0 | 0 | 0 | 0 | 0.01 | 0 | 0 | 0 | 0 | 0 | 0 | 0.01 | 0 | 0 | 0.01 | 0 | 0.05 | 0 | 0 | 0 | 0 | 0.03 | 0 | 0.13 | 0 | 0 | 0.08 | 0 | 0 | 0 | 0 | 0.10 | 0 | 0.06 | 0 | 0.05 | 0.13 |  |  |  |
| **49: BHS19** | 0 | 0 | 0 | 0 | 0 | 0 | 0 | 0 | -0.01 | 0 | -0.01 | -0.01 | -0.04 | 0 | 0 | 0 | 0.03 | 0 | 0 | 0 | -0.02 | 0 | 0 | 0 | -0.02 | 0 | 0 | 0 | 0.01 | 0.02 | 0.04 | 0 | 0.08 | -0.02 | 0 | 0.07 | 0 | 0.06 | 0 | 0.07 | 0 | 0 | 0.18 | 0 | 0.19 | 0 | -0.02 | -0.03 |  |  |
| **50: BHS20** | 0 | 0 | 0 | 0 | 0 | 0 | 0.02 | 0 | 0 | 0.01 | 0 | 0 | 0 | -0.01 | 0 | 0 | 0 | 0 | 0 | 0 | 0 | 0 | 0 | 0 | -0.01 | 0 | 0 | 0 | 0.01 | 0.04 | 0 | 0.02 | 0 | 0 | 0 | 0.02 | 0.07 | 0 | 0.11 | 0 | 0.10 | 0.03 | 0 | 0.09 | 0 | 0.20 | 0.19 | 0.23 | -0.02 |  |

**Supplemental Table 5** Edge weight of Network 2

|  | **1** | **2** | **3** | **4** | **5** | **6** | **7** | **8** | **9** | **10** | **11** | **12** | **13** | **14** | **15** | **16** | **17** |
| --- | --- | --- | --- | --- | --- | --- | --- | --- | --- | --- | --- | --- | --- | --- | --- | --- | --- |
| **1: SU1** |  |  |  |  |  |  |  |  |  |  |  |  |  |  |  |  |  |
| **2: SU2** | 0.43 |  |  |  |  |  |  |  |  |  |  |  |  |  |  |  |  |
| **3: SU3** | 0.18 | 0.16 |  |  |  |  |  |  |  |  |  |  |  |  |  |  |  |
| **4: SU4** | 0.07 | 0.13 | 0.33 |  |  |  |  |  |  |  |  |  |  |  |  |  |  |
| **5: SU5** | 0.10 | 0.07 | 0 | -0.02 |  |  |  |  |  |  |  |  |  |  |  |  |  |
| **6: SU6** | 0 | 0 | 0.03 | -0.02 | 0.60 |  |  |  |  |  |  |  |  |  |  |  |  |
| **7: SU7** | 0 | 0 | 0.02 | 0.05 | 0.07 | 0.19 |  |  |  |  |  |  |  |  |  |  |  |
| **8: SU8** | 0 | 0 | 0 | 0.13 | 0.11 | 0.19 | 0.36 |  |  |  |  |  |  |  |  |  |  |
| **9: SU9** | 0 | 0.05 | 0 | 0.09 | 0 | 0 | 0.04 | 0.09 |  |  |  |  |  |  |  |  |  |
| **10: SU10** | 0 | 0.02 | 0 | 0.06 | 0.01 | 0.04 | 0.06 | 0.08 | 0.61 |  |  |  |  |  |  |  |  |
| **11: SU11** | 0 | 0.06 | 0.15 | 0 | 0.04 | 0.01 | 0.05 | 0 | 0.09 | 0.12 |  |  |  |  |  |  |  |
| **12: SU12** | 0.01 | 0 | 0.06 | 0.12 | 0 | 0 | 0.05 | 0 | 0.16 | 0 | 0.54 |  |  |  |  |  |  |
| **13: SU13** | 0 | 0 | 0 | 0 | 0.01 | 0 | 0 | 0.02 | 0 | 0.02 | 0 | 0 |  |  |  |  |  |
| **14: SU14** | 0 | 0 | 0 | 0 | 0 | 0 | 0 | 0 | 0 | 0 | 0 | 0.02 | 0.31 |  |  |  |  |
| **15: SU15** | 0 | 0 | 0 | 0 | 0 | 0 | 0.01 | 0 | 0 | 0 | 0 | 0 | 0.21 | 0.41 |  |  |  |
| **16: SU16** | 0 | 0 | 0 | 0 | 0 | -0.02 | 0 | -0.01 | 0 | 0 | 0 | 0 | 0.08 | 0.22 | 0.49 |  |  |
| **17: SU17** | 0.01 | 0 | 0 | 0 | -0.01 | -0.02 | 0 | -0.02 | -0.02 | 0 | 0 | 0.01 | 0.01 | 0.05 | 0.03 | 0.08 |  |

**Supplemental Table 6** Edge weight of Network 3

|  | **1** | **2** | **3** | **4** | **5** | **6** | **7** | **8** |
| --- | --- | --- | --- | --- | --- | --- | --- | --- |
| **1: SigOther** |  |  |  |  |  |  |  |  |
| **2: Family** | 0.86 |  |  |  |  |  |  |  |
| **3: Friends** | 0.89 | 0.89 |  |  |  |  |  |  |
| **4: Gov** | 0.05 | 0.03 | 0.06 |  |  |  |  |  |
| **5: GAD 7** | -0.12 | -0.14 | -0.14 | -0.04 |  |  |  |  |
| **6: PHQ 15** | -0.08 | -0.08 | -0.10 | -0.06 | 0.68 |  |  |  |
| **7: PHQ 9** | -0.14 | -0.16 | -0.16 | -0.06 | 0.83 | 0.69 |  |  |
| **8: BHS** | -0.32 | -0.43 | -0.37 | 0.00 | 0.30 | 0.14 | 0.33 |  |
