# Supplementary material for: A network analysis of mental, somatic health, and perceived social supports among Chinese pregnant and postpartum women
Source: Glob Health Action. 2026 Feb 2;19(1):2615495. doi: 10.1080/16549716.2026.2615495 (PMC12865834; doi:10.1080/16549716.2026.2615495)
Supplement: STROBE.doc [file ZGHA_A_2615495_SM8556.doc]

STROBE Statement—Checklist of items that should be included in reports of ***cross-sectional studies***

|  | Item No | Recommendation | Page |
| --- | --- | --- | --- |
| **Title and abstract** | 1 | (*a*) Indicate the study’s design with a commonly used term in the title or the abstract | P3 Line 5-7 |
|  |  | (*b*) Provide in the abstract an informative and balanced summary of what was done and what was found | P3 Line 8-20 |
| Introduction | | |  |
| Background/rationale | 2 | Explain the scientific background and rationale for the investigation being reported | P4 Line 27-73 |
| Objectives | 3 | State specific objectives, including any prespecified hypotheses | P5 Line 74-81 |
| Methods | | |  |
| Study design | 4 | Present key elements of study design early in the paper | P6 Line 82-83 |
| Setting | 5 | Describe the setting, locations, and relevant dates, including periods of recruitment, exposure, follow-up, and data collection | P5 Line 84-94 |
| Participants | 6 | (*a*) Give the eligibility criteria, and the sources and methods of selection of participants | P6 Line 92-94 |
| Variables | 7 | Clearly define all outcomes, exposures, predictors, potential confounders, and effect modifiers. Give diagnostic criteria, if applicable | P6 Line 95-144 |
| Data sources/ measurement | 8* | For each variable of interest, give sources of data and details of methods of assessment (measurement). Describe comparability of assessment methods if there is more than one group | P8 Line 145-171 |
| Bias | 9 | Describe any efforts to address potential sources of bias | P8 Line 172-178 |
| Study size | 10 | Explain how the study size was arrived at | P6 Line 91-94 |
| Quantitative variables | 11 | Explain how quantitative variables were handled in the analyses. If applicable, describe which groupings were chosen and why | P6 Line 95-144 |
| Statistical methods | 12 | (*a*) Describe all statistical methods, including those used to control for confounding | P8 Line 145-171 |
|  |  | (*b*) Describe any methods used to examine subgroups and interactions | NA |
|  |  | (*c*) Explain how missing data were addressed | P6 Line 93 |
|  |  | (*d*) If applicable, describe analytical methods taking account of sampling strategy | NA |
|  |  | (*e*) Describe any sensitivity analyses | NA |
| Results | | |  |
| Participants | 13* | (a) Report numbers of individuals at each stage of study—eg numbers potentially eligible, examined for eligibility, confirmed eligible, included in the study, completing follow-up, and analysed | P6 Line 92 |
|  |  | (b) Give reasons for non-participation at each stage | NA |
|  |  | (c) Consider use of a flow diagram | NA |
| Descriptive data | 14* | (a) Give characteristics of study participants (eg demographic, clinical, social) and information on exposures and potential confounders | P9 Line 179-181 |
|  |  | (b) Indicate number of participants with missing data for each variable of interest | NA |
| Outcome data | 15* | Report numbers of outcome events or summary measures | P9 Line 182-187 |
| Main results | 16 | (*a*) Give unadjusted estimates and, if applicable, confounder-adjusted estimates and their precision (eg, 95% confidence interval). Make clear which confounders were adjusted for and why they were included | NA |
|  |  | (*b*) Report category boundaries when continuous variables were categorized | NA |
|  |  | (*c*) If relevant, consider translating estimates of relative risk into absolute risk for a meaningful time period | NA |
| Other analyses | 17 | Report other analyses done—eg analyses of subgroups and interactions, and sensitivity analyses | P9 Line 188-236 |
| Discussion | | |  |
| Key results | 18 | Summarise key results with reference to study objectives | P11 Line 237-247 |
| Limitations | 19 | Discuss limitations of the study, taking into account sources of potential bias or imprecision. Discuss both direction and magnitude of any potential bias | P13 Line 314-321 |
| Interpretation | 20 | Give a cautious overall interpretation of results considering objectives, limitations, multiplicity of analyses, results from similar studies, and other relevant evidence | P11 Line 248-299 |
| Generalisability | 21 | Discuss the generalisability (external validity) of the study results | P13 Line 300-313 |
| Other information | | |  |
| Funding | 22 | Give the source of funding and the role of the funders for the present study and, if applicable, for the original study on which the present article is based | P20 |

*Give information separately for exposed and unexposed groups.

**Note:** An Explanation and Elaboration article discusses each checklist item and gives methodological background and published examples of transparent reporting. The STROBE checklist is best used in conjunction with this article (freely available on the Web sites of PLoS Medicine at http://www.plosmedicine.org/, Annals of Internal Medicine at http://www.annals.org/, and Epidemiology at http://www.epidem.com/). Information on the STROBE Initiative is available at www.strobe-statement.org.
